# Markerless 3D Skeleton Tracking Algorithm by Merging Multiple Inaccurate Skeleton Data from Multiple RGB-D Sensors

**DOI:** 10.3390/s22093155

**Published:** 2022-04-20

**Authors:** Sang-hyub Lee, Deok-Won Lee, Kooksung Jun, Wonjun Lee, Mun Sang Kim

**Affiliations:** School of Integrated Technology, Gwangju Institute of Science and Technology, Gwangju 61005, Korea; sang-hyub@gist.ac.kr (S.-h.L.); deokwon0915@gm.gist.ac.kr (D.-W.L.); kooksung930@gm.gist.ac.kr (K.J.); leewonjun@gm.gist.ac.kr (W.L.)

**Keywords:** skeleton tracking, sensor fusion, multiple RGB-D sensors, motion capture

## Abstract

Skeleton data, which is often used in the HCI field, is a data structure that can efficiently express human poses and gestures because it consists of 3D positions of joints. The advancement of RGB-D sensors, such as Kinect sensors, enabled the easy capture of skeleton data from depth or RGB images. However, when tracking a target with a single sensor, there is an occlusion problem causing the quality of invisible joints to be randomly degraded. As a result, multiple sensors should be used to reliably track a target in all directions over a wide range. In this paper, we proposed a new method for combining multiple inaccurate skeleton data sets obtained from multiple sensors that capture a target from different angles into a single accurate skeleton data. The proposed algorithm uses density-based spatial clustering of applications with noise (DBSCAN) to prevent noise-added inaccurate joint candidates from participating in the merging process. After merging with the inlier candidates, we used Kalman filter to denoise the tremble error of the joint’s movement. We evaluated the proposed algorithm’s performance using the best view as the ground truth. In addition, the results of different sizes for the DBSCAN searching area were analyzed. By applying the proposed algorithm, the joint position accuracy of the merged skeleton improved as the number of sensors increased. Furthermore, highest performance was shown when the searching area of DBSCAN was 10 cm.

## 1. Introduction

### 1.1. Research Background

The captured human pose or gestured data can provide a lot of useful information for developing human–robot interaction (HRI) or human action recognition. The skeleton data, which consists of human joint position, is one of the most commonly used pieces of information in human research. This is because the number of joints that comprise the skeleton does not vary according to the shape of the human body or gender, maintaining a standardized structure that represents the human pose.

Due to these advantages, skeleton data is widely used in developing games for humans, industrial environments, and research on pathological diagnosis through recognition of gestures. M. Ma, et al. [1] developed a multi-planar full-body rehabilitation game named *Mystic Isle* using Microsoft Kinect V2. The user can interact with virtual environment by using their body. The Kinect V2 sensor tracked the user’s body, and the body tracking SDK provided the tracked skeleton data, which included 25 joints. A. Taha et al. [2] attempted to obtain descriptive labeling of complex human activities using Kinect V2 skeleton data. They proposed building the specific feature vector using identified skeleton joint coordinates as input for the Hidden Markov model.

In [3], M. Varshney, et al. proposed the rule-based classifier method that recognizes a view-invariant multiple human activity recognition in real time. They also used a single Kinect V2 sensor and body tracking SDK to track the human skeleton data. The skeleton data was then used as input for the proposed classifier method. E. Cippitelli et al. [4] attempted to recognize human activity using skeleton data obtained from RGB-D sensors. They also extracted a specific feature vector from skeleton pose data and used a support vector machine to classify human actions. Bari proposes a gait recognition model based on deep learning architecture in his paper [5]. They also trained the suggested deep learning model using the public 3D skeleton gait database recorded with the Microsoft Kinect V2 sensor. Based on skeleton data, two unique geometric features, the joint relative cosine dissimilarity and joint relative triangle area, are constructed. Consequently, the skeleton data is utilized in wide research areas that target human gesture or interaction as the raw or input data. Skeleton-based approaches, on the other hand, do not always ensure good performance. If the skeleton data is incorrect or noisy, they perform poorly. In other words, the quality of the skeleton data may be a determinant of the performance of the human tracking system or gesture recognition model [6].

To capture the human gesture using skeleton data accurately, the typical system is the marker-based motion capture system such as VICON (Vicon Motion Systems Ltd., Oxford, UK) and Opti-track (Natural Point Inc., St Corvallis, OR, USA) because of their proven accuracy. These systems require wearing a suit attached with a reflective marker or a process for attaching the marker to the human body. This procedure takes a long time, and the attached marker makes the human’s movement unnatural [7]. Therefore, the motion capture system is difficult to apply in a variety of environments for capturing human gestures.

**Figure 1:**
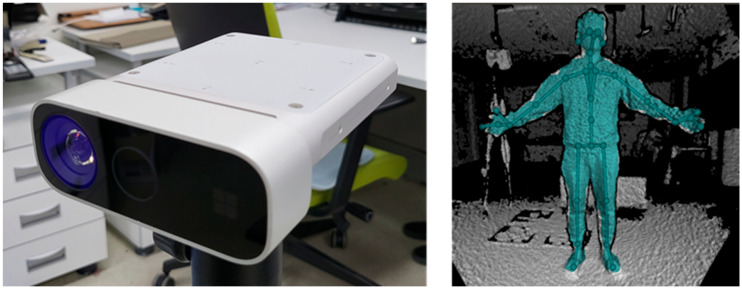
(**Left**), Azure Kinect and (**Right**), body tracking SDK of Azure Kinect.

In many recent studies, a human motion capturing system using an RGB-Depth (RGB-D) sensor such as Kinect (Microsoft Corp., Redmond, WA, USA), Xtion (ASUS, Taipei, Taiwan), Astra (Orbbec 3D Technology International, Inc., Troy, MI, USA), or Realsense (Intel Corp., Santa Clara, CA, USA) that does not require marker attachment is widely used as an alternative method [8,9,10]. The MS Kinect sensor is the most widely used in motion capture research, and it includes not only the sensor SDK, which captures RGB and depth data, but also the body tracking SDK, which captures 3D skeleton data from the depth image [11]. The Azure Kinect sensor is the most recent addition to the Kinect sensor series. The Azure Kinect body tracking SDK provides skeleton data consisting of 3D positional information for 32 joints, as shown in Figure 1. The random forest algorithm is adopted in Kinect v2 whereas a deep learning-based algorithm is adopted to Azure Kinect’s body tracker [12,13]. The GPU can be used to run the deep learning model extracting human skeleton data. Furthermore, because the sensor SDK has been upgraded to allow multiple sensors to be operated on a single PC, it can be effectively applied in a variety of research or industrial areas.

Although skeleton tracking for a whole body is possible using Azure Kinect, poor skeleton quality often occurs due to a problem called self-occlusion [14]. This problem is a limitation of the single sensor system and occurs when the target joint is obscured by other body parts. The issue causes the quality of skeleton data to degrade at random. One of the simplest ways to solve this problem is to use a motion capture system with multiple sensors that can cover the entire workspace area [15,16]. For example, if an occlusion problem causes an error in the skeleton data, the inaccurate information of the obscured joint can be compensated by using information from another sensor. In other words, a single sensor’s self-occlusion problem can be overcome by appropriately combining skeleton data from multiple sensors.

In this paper, we developed a new algorithm that merges the multiple skeleton data obtained by multiple RGB-D sensors in real time. We used Azure Kinect sensors of Microsoft because of the sensor’s convenient expandability meaning that the multiple sensors can be utilized on a single PC. The skeleton data was obtained using the Azure Kinect body tracking SDK. Because the Azure Kinect sensor provides a function that can synchronize time steps between sensors by linking them together, no additional work for time synchronization is required when using multiple sensors. Specifically, we adopted the density-based spatial clustering of applications with noise (DBSCAN) which is generally used for clustering as the error filter on the skeleton merging process. After the merging process, we used the Kalmal filter to minimize the tremble error in joint movements.

There are three main contributions. First, the proposed algorithm can merge the skeleton data accurately in real time. We demonstrated how the number of sensors (TNOS) increased the joint accuracy of the merged skeleton. Second, the error caused by self-occlusion is avoided during the merging process of skeletons obtained by multiple Kinects using DBSCAN, a clustering algorithm. Third, we reduced joint position tremble error by using a Kalman filter on merged skeleton data. With these contributions, the proposed method can help improve the performance of various skeleton-based research and applications by obtaining accurate skeleton data in all directions.

### 1.2. Related Works

There have been many studies dealing with markerless skeleton tracking to overcome the difficult usability of motion capture equipment. Among them, studies using RGB-D sensors are representative. The development of many kinds of RGB-D sensors provides the human pose information in the form of the skeleton data extracted from depth images. However, there is a problem known as self-occlusion, which is caused by the sensor’s limited viewing area. Furthermore, when the subject is facing the sensor, the skeleton tracking algorithm can provide the best accuracy [17]. In other words, the subject can be tracked more reliably when facing the sensor in a pose with no invisible joint [14].

To address the occlusion issue, several studies adopted the multiple RGB-D sensors system to minimize invisible body parts. They made several attempts to obtain optimized skeleton data by combining multiple skeleton data obtained from multiple sensors installed in various views. Several studies have been conducted to merge multiple skeleton data sets using constraint rules determined by the structure of the human skeleton. These attempts were conducted by assigning different weights to inaccurate joint candidates in the merging process. These types of trials may necessitate an initial configuration process to determine structural components such as bone length.

Y. Kim, et al. [18] proposed a motion capture system using multiple Kinect V2 sensors for capturing the dynamic gestures of humans in a 3D environment. A posture reconstruction method was adopted for tracking human gestures consistently. They proposed a tracking method for the torso joints and limb joints separately, based on the consistent bone length of the human body. The mean value of the candidates within the bone length in the direction from the parent joint to the target joint was calculated in the case of the torso. In the case of limb, a joint candidate with the smallest sum of rotation direction and rotation angle compared to the previous joint coordinates was chosen from among the candidate groups within the bone length threshold. J. Colombel et al. [19] presented a fusion algorithm for tracking the joint’s center position using multiple skeleton data from multiple depth cameras to improve human motion analysis. The proposed system adopted an extended Kalman filter for the fusion of the joint candidates into the joint center position and applied the anthropomorphic constraints of human skeleton structure. As the measurement model of the extended Kalman filter, a specific forward kinematics model representing the human locomotor system with fixed bone lengths was used. The measurement fusion method was chosen among the fusion methods based on the Kalman filter because the proposed algorithm should be run in real time. N. Chen et al. [20] describe a method for combining two skeleton data sets from two Kinect V2 sensors. They proposed a data fusion strategy that weights the candidate of the target joint based on human physiological movement constraints related to both bone length and joint angle.

The other approach for the development of a multiple skeleton fusion algorithm is the definition of confidence value for a joint candidate in the merging process. The confidence value is usually determined according to the state in which the joint or skeleton data is detected in the sensor’s view. In [21], the authors proposed the human pose estimation method by fusing the multiple skeleton data and tracking the merged skeleton data. They considered the confidence value at both the whole skeleton and each joint level, and they filtered the inaccurate skeleton or joint data by using a confidence value threshold. Finally, the fused skeleton was tracked using the Kalman filter. Y. Wu et al. [22] created a real-time full-body tracking system with three Kinect V2 sensors. They used an adaptive weighting adjustment fusion method to build merged 3D skeleton data regardless of the subject’s orientation. Each candidate of the target joint obtained from each sensor was weighted according to the angle between sensor and subject and participated in the merging process. K. Desai and S. Raghuraman [23] proposed a real-time skeletal pose tracking method that aims to get merged skeleton data using multiple inaccurate skeletons. They determined a new confidence value named probability of an accurate joint (PAJ) for each target joint. Several factors were considered when determining the PJA. First, PAJ was calculated using the skeleton’s orientation, which is the angle between the subject’s facing direction and the sensors. The second state is the joint state, which indicates that the target joint is visible in the sensor’s visible area. The third factor is the bone angle, which is calculated between the bone and the capture plane, as well as the fixed length of a human’s bone. Taking into account all components, the merged skeleton’s joint position was determined using a distance-constrained consensus approach that maximizes the overall PAJ.

Some studies tried to design a new merging process for calculating the position of a joint. Moon and others [24] developed a human skeleton tracking system using Kalman filter framework with weighted measurement fusion method for merging five inaccurate skeleton data. The five Kinect V2 sensors were used to capture the subject, and the measurement noise of the Kalman filter was controlled based on the predicted state and joint motion continuity. In addition, H. Zhang et al. [25] proposed a method for combining multiple skeleton data sets. The proposed method’s first strategy was to filter outliers among target joint candidates using spatial region constraints and K-means clustering. The second step was to combine the inlier candidates into a single skeleton and apply the proposed adaptive weighted fusion rules. K. Ryselis et al. [26] presented a practical solution for performing multiple skeleton data fusion algorithms in vector space using algebraic operations. They aimed to track the human with non-standard poses such as squatting, sitting, and lying.

As in the studies described above, the algorithm developed in this paper does not estimate the skeleton using raw data such as RGB, Depth, and Pointcloud, but creates optimized skeleton data by merging multiple inaccurate skeleton data measured from various angles. In particular, as in [25], we also propose a method of filtering inaccurate joint candidates by applying a clustering algorithm in the merging process.

The remainder of the paper is structured as follows: Section 2 describes all sensor’s coordinate system calibration methods, a proposed merging algorithm that uses DBSACN, and settings of our experiment; Section 3 describes the result of the experiment; Section 4 discusses the experimental result and the future works of this study. Finally, we present our conclusions in Section 5.

## 2. Materials and Methods

### 2.1. Calibration for Coordinate Systems of Sensors

**Figure 2:**
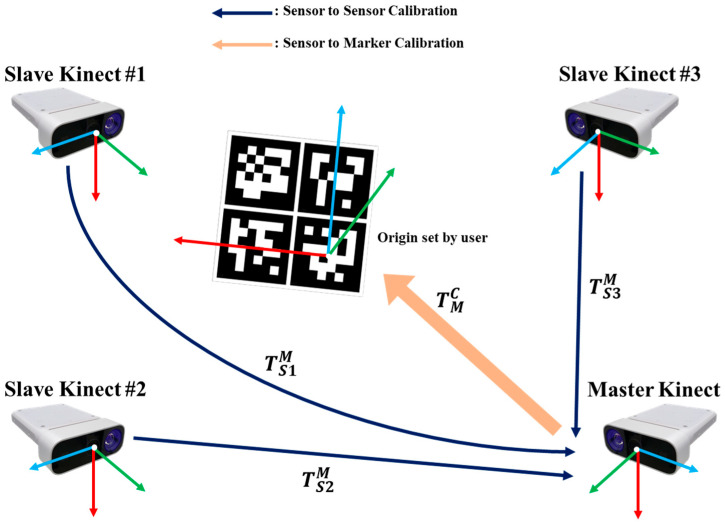
Proposed calibration procedure.

To use the 3D data acquired using multiple RGB-D sensors, a calibration process must be performed first. Moreover, since the calibration accuracy can have a great effect on the joint position of the merged skeleton data, an accurate calibration process must be performed. The calibration process refers to matching the coordinate systems of each sensor into one global coordinate system by calculating a rigid transformation matrix $M=\left[R,\;T\right]$. Here, R is rotation matrix parameterized by the three rotations $\theta_{x}$, $\theta_{y}$, and $\theta_{z}$. Additionally, *T* is a translation matrix consisting of three translation offset values for the *x*, y, and z axes, respectively. In this paper, two steps for the calibration process were adopted as described in Figure 2. The first one is sensor-to-sensor calibration matching the coordinate system of each sensor to the coordinate system of the reference sensor set as a master sensor. Another is the sensor-to-marker calibration resetting the coordinate system of all sensors calibrated with the reference sensor to the global origin customized by the user.

#### 2.1.1. Sensor-to-Sensor Calibration

**Figure 3:**
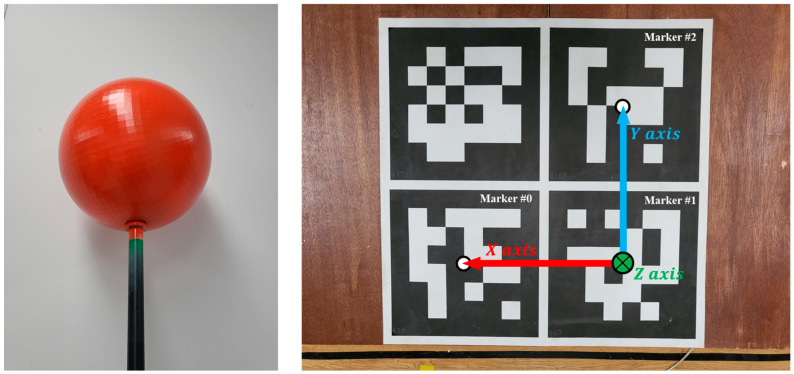
(**Left**), sphere object and (**Right**), plane marker.

The sensor-to-sensor calibration process was conducted by constructing correspondence trajectories composed of the 3D centroid points of the sphere object. Figure 3 depicts a sphere object with a radius of 24 cm and a red tone color. The RGB image was first converted into HSV color space to track the center coordinates of a spherical object. Additionally, the histogram backprojection algorithm [27] was used to extract the area of a specific hue value. The Pointcloud Data (PCD) corresponding to the sphere object is then obtained by extracting the pixels in the depth image that correspond to the pixels in the HSV image that correspond to the sphere. Finally, the RANSAC [28] algorithm identified iteratively all the 3D points that satisfy the surface equation of the sphere object with radius R to detect the robust 3D centroid points of sphere object. The 3D centroid points of the sphere object extracted for several frames from each sensor constitute a correspondence trajectory. In the process of capturing the sphere, frames in which one or more sensors do not detect the object are filtered out in the construction trajectory.

To obtain a rigid transformation matrix between the coordinate systems of the master and target sensors, singular value decomposition (SVD) was calculated on a pair of trajectories configured in the corresponding sensors [29]. Let the $C^{M}$ be the trajectory captured in the coordinate system of the master sensor and $C^{p}$ be the trajectory captured in the coordinate system of target sensor number p, we have the rigid transformation matrix RT between $C^{p}$ and $C^{M}$ as:

(1) $$RT^{\left(Mp\right)}=\left(\begin{array}{cc} R^{\left(pM\right)} & T^{\left(pM\right)}\\ 0 & 1 \end{array}\right)$$

To compute unknown parameters of the $R^{pM}$, $T^{pM}$ matrix, the trajectory with 3 or more correspondences is required. In this study, we collected 1000 points of correspondence to create a trajectory. The optimal transformation matrix could then be computed by minimizing the error function shown below.

(2) $$E(R^{\left(pM\right)},T^{\left(pM\right)})\;\propto \;\overset{N}{\underset{i=1}{\sum }}\|C_{i}^{M}-{\left(R^{\left(pM\right)}C_{i}^{p}+T^{\left(pM\right)})\right\|}^{2}$$

where N is the number of correspondences of the trajectory. To compute the rotation matrix, we used the least-squares-based method described in [30]. Thus, the optimal solution for $E\left(R^{\left(pM\right)},\;T^{\left(pM\right)}\right)$ could be obtained by computing the covariance matrix as follows:

(3) $$Cov=\overset{N}{\underset{i=1}{\sum }}[\left({\hat{C}}^{p}-C_{i}^{p})\cdot ({\hat{C}}^{M}-C_{i}^{M}\right)]$$

where, ${\hat{C}}^{i}$ is the centroid of trajectory captured on the coordinate system of target sensor number p, and ${\hat{C}}^{M}$ is the centroid of trajectory captured on the coordinate system of the master sensor. By using SVD, the covariance matrix Cov  is decomposed as $Cov=USV^{T}$. Then, the rotation matrix could be calculated as $R^{\left(pM\right)}=UV^{T}$ and translation matrix $T^{\left(pM\right)}={\hat{C}}^{M}-{\hat{C}}^{p}$. This procedure occurs between all coordinate systems of target sensors and the master sensor; as a result of this procedure, all coordinate systems of target sensors are calibrated with the coordinate system of the master sensor.

#### 2.1.2. Sensor-to-Marker Calibration

In this paper, we performed marker calibration to obtain the collected 3D skeleton data based on the desired coordinate system. This is accomplished by placing a plane marker with a specific pattern in the capture area and recognizing it in master Kinect’s RGB view. As shown in Figure 3, we used a plate printed with four ARUCO markers. The method of [31] implemented in the OpenCV ver3.4.0 library was used for marker recognition. After detecting the marker in the master Kinect’s RGB image, the PCD corresponding to each marker can be extracted from the depth image collected along with the RGB image. Then, the centroid of the PCD is calculated to obtain the three-dimensional center points of all markers.

To set all coordinate systems to a custom coordinate system defined by the user, we calculate a rigid transformation matrix by setting the desired coordinate axis and origin point using the center points. As shown in Figure 3, the vector between the centroid of marker number 2 and the centroid of marker number 1 serves as the x-axis in this study, while the vector between the centroid of marker number 0 and the centroid of marker number 1 serves as the y-axis. The z-axis was set in the direction from the floor to the ceiling by calculating the cross-product of the x and y axes, and the origin point was set as the centroid of marker number 1.

After sensor-to-marker calibration, the coordinate systems of all target sensors transformed to the master sensor are transformed again to the custom coordinate system. Throughout this procedure, all data obtained from multiple RGB-D sensors can be taken based on the desired coordinate system and origin set by the user.

### 2.2. Skeleton Merging Algorithm

**Figure 4:**
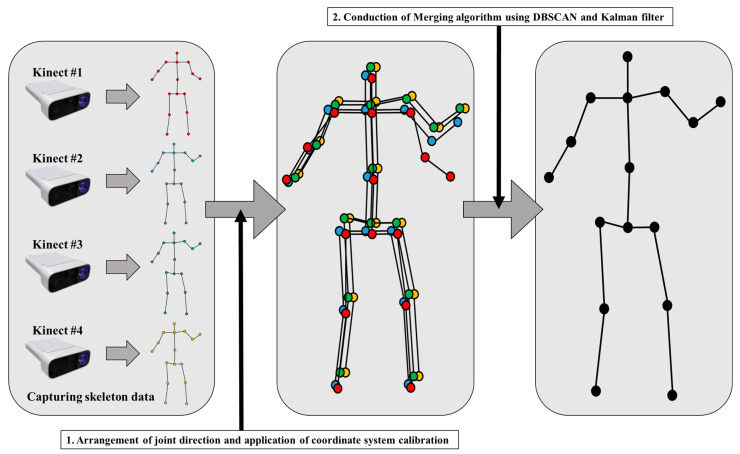
Proposed skeleton merging algorithm.

As mentioned in Section 1, a self-occlusion problem that randomly degrades the quality of the skeleton data could occur when using a single RGB-D sensor to track the target. For handling this issue, we adopted multiple RGB-D sensors to overcome the self-occlusion problem. If a joint in a position invisible to one sensor is visible to another, it will be possible to compensate using the appropriate merging algorithm. The main issue with tracking skeletons with multiple sensors is figuring out how to combine multiple inaccurate skeleton data sets. In this paper, we designed a skeleton merging algorithm that increases the accuracy of the merged skeleton according to the TNOS, as shown in Figure 4. After capturing skeleton data and applying calibration, the merging process is applied. The proposed algorithm’s first component is the rearrangement of a joint’s directions, and the second is DBSCAN, which is used as a noise filtering method in the merging process [32]. Lastly, the third one is the tracking method based on Kalman filter to track the target joint and make the movement of joint smooth by canceling the tremble error.

#### 2.2.1. Arrangement of Skeleton to Correct the Misoriented Joints

**Figure 5:**
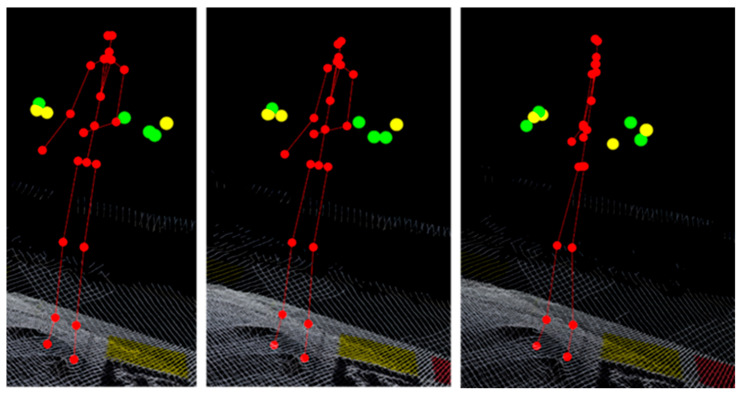
Three different examples of a misorientation error situation (yellow sphere: the candidate positions of the right elbow; green: the candidate positions of the left elbow).

Before we apply the merging process, the arrangement procedure that corrects the direction of joints is needed because there is a misoriented problem mentioned in [21,23,33]. In more detail, the misoriented problem refers to a phenomenon in which the left–right direction of the joint in the measured skeleton data differs from the actual joint’s left–right direction. For example, while one of the measured skeleton’s joints is labeled as the left shoulder, for the actual skeleton it may be the right shoulder. In addition, the left and the right directions of each joint may be recognized differently for each sensor. In Figure 5, three examples of misoriented problems are described. The yellow and green spheres indicate the positions of the elbow joint recognized by the four sensors as left and right, respectively. Since the spheres of the same color are not adjacent to each other but are mixed, it is confirmed that a misoriented problem occurs in the skeleton data obtained by the SDK. As a result, if joints are merged without alignment, it is inevitable to obtain crushed skeleton data such as the red skeleton in Figure 5.

For that problem, the arrangement process for each of the left and right joint sets is adopted. The body tracking SDK of Azure Kinect provides the confidence value of tracked joints. There are three levels of confidence values: medium, low, and none. If the tracker can track the joint with an average level of confidence, the joint will be assigned a medium level of confidence value. Additionally, the low level is assigned to a joint that is not tracked but is estimated by the tracker because it is occluded or invisible. The joint with the none level indicates that the target joint is not in the field of view. Additionally, this level of assurance was used as the arrangement’s standard.

At first, for each of the left and right joints, the reference position for arrangement is set by the average point of the joint positions that have a medium confidence level (if there is no medium level joint, the standard will be low level). After, the distance comparing will be conducted. Let $P_{i}^{Rr}=\left[x_{i}^{Rr},\;y_{i}^{Rr},\;z_{i}^{Rr}\right],\;\;P_{i}^{Rl}=\left[x_{i}^{Rl},\;y_{i}^{Rl},\;z_{i}^{Rl}\right]$ where the $P_{i}^{Rr},\;P_{i}^{Rl}$ are the reference positions of the right and left joint, respectively. Additionally, i is the number of the set of left and right joints that should be verified. Then, the distance between the reference position and the joints in both directions is calculated and compared to correct the misoriented problem as the following function.

(4) $$D_{i}^{correct}=Dist\left(P_{i}^{Rr},\;P_{i}^{Tr}\right)\;$$

(5) $$D_{i}^{wrong}=Dist\left(P_{i}^{Rr},\;P_{i}^{Tl}\right)$$

Here, the $Dist\left(P^{1},P^{2}\right)$ means the Euclidian distance (mm) between $P^{1}$ and $P^{2}$, and the $P_{i}^{Tr},\;P_{i}^{Tl}$ are the positions of the target joint of the right and left sides, respectively. Then, we defined the checking rules to detect the misoriented problem as:

(6) $$\left\{\begin{array}{c} D_{i}^{correct}>D_{i}^{wrong}\;\;problem\\ D_{i}^{correct}<D_{i}^{wrong}\;\;no\;problem \end{array}\right.\;$$

When the misoriented joint set is detected, the direction label of the target joint set composed of left and right is corrected. The Azure Kinect body tracking SDK was used for this study, and the tracker provided skeleton data with 32 joints. However, we only used 16 joints from the torso (pelvis, spine, chest, neck, head, and shoulder) and limbs (elbow, wrist, hip, knee, and ankle). This is because other joints do not have high accuracy, including a large error when the subject performs a large movement action. The proposed arrangement process is applied to all limb joints, hip joints, and shoulder joints in this study to correct misaligned joints.

#### 2.2.2. Skeleton Merging and Noise Filtering Using DBSCAN

**Figure 6:**
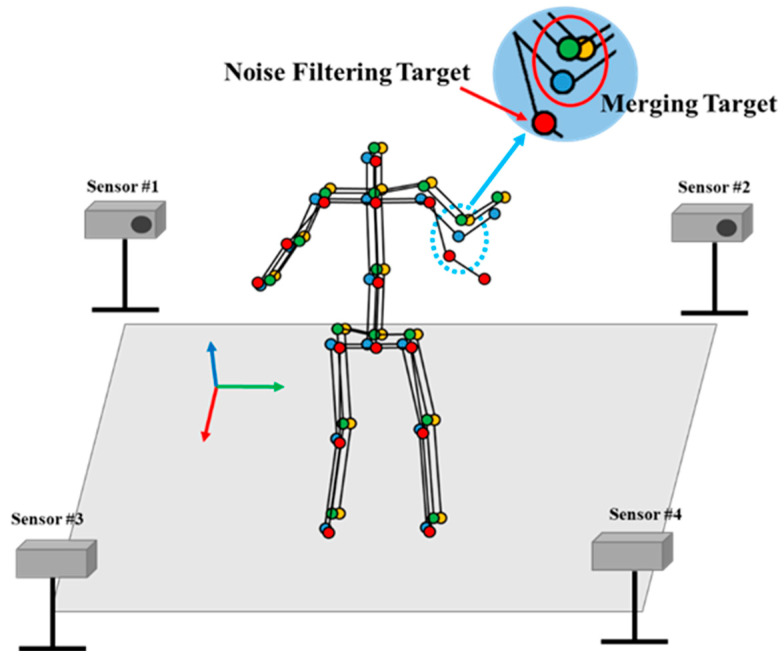
DBSCAN for joint merging.

In the merging process, we tried to conduct the merging process with only inlier candidates by noise filtering based on the positions of candidates. Noise candidates were defined as incorrectly recognized joints collected from sensors that had issues recognizing target joints. When compared to the inlier candidate, the noise candidate is relatively far from the actual target joint position. In this study, we use DBSCAN to filter these noise candidates during the merging process, as shown in Figure 6.

DBSCAN is a clustering method based on the density of target data distribution. It operates under the assumption that candidates belonging to the same cluster will be distributed close to each other. Because it operates by including adjacent data in the same cluster based on the density of data, DBSCAN can perform clustering well on data of unspecified shapes. Furthermore, DBSCAN can classify noise data while clustering, which mitigates the degradation of clustering performance caused by outlier participation. As a result of merging joint candidates that can be randomly distributed, noise can be appropriately filtered by using DBSCAN. It operates with hyperparameters composed of the neighboring data searching area ∈ and the minimum number of neighboring data $N_{c}$. The detailed operation procedure of DBSCAN is described in Algorithms 1 and 2.
**Algorithm 1: DBSCAN****Input:**candidates set χ,
searching area ϵ,
minimum number of neighboring data $N_{c}$**Output:**labels yk=0 // number of cluster**foreach**x∈χ**do**
 $y_{x}$ ← UNASSIGNED**end****foreach**x∈χ**do**
 **if**
$y_{x}$ = UNASSIGNED **then**

  $\chi_{x}$ = SCAN(x, ϵ) // Searching neighbors of x
  **if**
$\left|\chi_{x}\right|\;\geq \;N_{c}$
**then**
   k
*←*
k+1
   $y_{x}$
*←*
k
    **foreach**
$z\in \chi_{x}$
**do**
    **if**
$y_{z}=$ UNASSIGNED **then**
     $y_{z}$
 ← k
      $\chi_{z}$ = SCAN(x, ϵ)
      **if**
$\left|\chi_{z}\right|\;\geq \;N_{c}$
**then**       $\chi_{x}$ ← $\chi_{x\;}\cup \chi_{z}$
      **end**
     **end**
    **end**
  **end**
  **else**
   $y_{x}$ ← NOISE
  **end**
 **end****end****return**y


**Algorithm 2: SCAN**

**Input:**
data point χ,
searching area ϵ
**Output:**
neighbors $\chi_{x}$**foreach**z∈χ**do**
 **if**
*Euclidean_distance*(x, z) ≤
ϵ
**then**
  $\chi_{x}$ ← $\chi_{x}\cup \;z$
 **end**
**end**
**return**
$\chi_{x}$

Our proposed algorithm defines the probability that the data in the cluster are the same as the actual location of the target joint considering both the number and the density of the cluster. In other words, it is assumed that the more densely the positions of candidate joints recognized from various angles belong, the higher the probability that the data constituting the cluster is the same as the actual joint coordinates. We use two more tricks in this case. First, the candidate for the reference position mentioned in Section 2.2.1 was chosen. In the clustering process, it gives more weight to candidates with a high confidence value of tracking state. Second, the previous position of the target joint was included as a clustering candidate. Even if an occlusion problem occurs during the skeleton data recognition process, the noise may be too large for DBSCAN to filter. Furthermore, there are cases where the movement of the recognized joint exceeds the actual joint movement distance or there is completely no recognized joint movement. To solve this problem, we added a smoothing effect to the movement of the joint by including the previous joint position in the clustering process.

After applying DBSCAN, we selected the cluster containing the most points as the candidate group for the target joint. Furthermore, we used the centroid of the chosen cluster as the merged joint position. The reference position and previous coordinates of the target joint, which were also included, were not used for the centroid calculation at this time to ensure the range of the actual joint movement. Among the hyperparameters of DBSCAN described above, $N_{c}$ was fixed to 1. In addition, the neighboring data searching area ∈ cannot be specified. Therefore, we conducted experiments on the searching areas of 5, 10, 15, and 20 cm in Section 3.

#### 2.2.3. Joint Position Tracking Using Kalman Filter

Even after the merging process, the tremble noise remains in the target joint. Therefore, we applied a Kalman filter-based tracking method to each joint to make the movement of the target joint smooth [34]. We denote $X_{t}^{j}=\left(X_{x,t}^{j}\;,\;\;X_{y,t\;}^{j},\;\;X_{z,t}^{j}\right)$ the state vector of the joint number j at time step t and $Z_{t}^{j}=\left(x_{Z,t\;}^{j},\;\;y_{Z,t}^{j}\;,\;\;z_{Z,t}^{j}\right)$ is the measurement vector for this joint resulting from the previously described merging algorithm. Then, we designed a linear system for a state model with process noise, and its measurement model with measurement noise as follows:

(7) $$X_{t+1}^{j}=AX_{t}^{j}+w_{p},\;w_{p}\;\sim \;N\left(0,\;Q_{t}\right)$$

(8) $$Z_{t}^{j}=HX_{t}^{j}+v_{m},\;v_{m}\;\sim \;N\left(0,\;R_{t}\right)$$

where A is the so-called state space transition model, H is the measurement matrix, and $w_{p}$ and $v_{m}$ are the process noise and measurement noise, respectively. Here, $w_{p}$ and $v_{m}$ are the white noise that complies with the Gaussian normal distribution with zero mean and covariance $Q_{t}$ and $R_{t}$, respectively. In our system, $Q_{t}$ and $R_{t}$ are set to 0.01 and 1.0, respectively.

For the input argument $X_{t}^{j}$, the 3D coordinate data of the target joint is used, and $Z_{t}^{j}$ is a corrected position of $X_{t_{t}}^{j^{j}}$ from the measurement step. The designed state model estimates a predicted position of joint, ${\tilde{X}}_{t}^{j}$, through the prediction and correction steps of the Kalman filter with $X_{t}^{j}$. In detail, the measurement step removes the noise in $X_{t}^{j}$ and then the prediction step estimates ${\tilde{X}}_{t}^{j}$. The predicted state vector ${\tilde{X}}_{t}^{j}$ and the predicted covariance matrix ${\tilde{P}}_{t}^{j}$ are estimated in the prediction step at the time step t−1 as follows:

(9) $${\tilde{X}}_{t}^{j}=A{\hat{X}}_{t-1}^{j}\;$$

(10) $${\tilde{P}}_{t}^{j}=AP_{t-1}^{j}A^{T}+Q_{t}\;$$

where ${\hat{X}}_{t-1}^{j}$ and ${\tilde{P}}_{t-1}^{j}$ are the posteriori state estimate and the posteriori error covariance matrix at the time step t−1, respectively. Here, ${\tilde{X}}_{t}^{j}$ is used as the tracked target joint position in this study. For the correction step, ${\tilde{X}}_{t}^{j}$*,* ${\tilde{P}}_{t}^{j}$, and the Kalman gain $K_{t}$ are used to calculate the posteriori system estimate ${\hat{X}}_{t}^{j}$ which removes the noise. Thus, ${\hat{X}}_{t}^{j}$ was calculated as the corrected position by using the measurement value $p_{t}^{j}$ and the posteriori covariance matrix $P_{t}^{j}$ as follows:

(11) $$K_{t}=\frac{{\tilde{P}}_{t}^{j}H^{T}}{H{\tilde{P}}_{t}^{j}H^{T}+R_{t}}\;$$

(12) $${\hat{X}}_{t}^{j}={\tilde{X}}_{t}^{j}+K_{t}\left(p_{t}^{j}-H{\tilde{X}}_{t}^{j}\right)\;$$

(13) $$P_{t}^{j}=\left(1-K_{t}H\right){\tilde{P}}_{t}^{j},$$

which will be used for the prediction step at time step t+1. By applying a Kalman filter to each joint and performing a tracking method, it is possible to obtain the smooth movement of joints with unrecognized movement or tremors.

### 2.3. Experiment Setting

In this section, the experiment settings and environment are described. All experiments were conducted on an Intel 19-11900F octa-core microprocessor clocked at 2.50 Ghz with 32 GB RAM. Additionally, we used two GeForce RTX 2060 super GPU for operating the Azure Kinect body tracking SDK. In the experiment, five Kinect Azure sensors were used to capture all of the subjects’ gestures. The sensor SDK version was 1.4.1, and the body tracking SDK version was 1.1.0. Additionally, every procedure was developed in the C++ environment. The proposed algorithm’s goal is to track skeleton data in real-time. However, when the body tracking SDK for five sensors was operated on two GPU, the capturing speed was less than 10 frames per second. Therefore, we chose the lite-model that had a 2 times performance increase and 5% accuracy decrease among the models of body tracking SDK (as described in https://docs.microsoft.com/en-us/azure/kinect-dk/ accessed on 17 April 2022). Additionally, the depth mode of the sensor was narrow field-of-view that has the smallest depth image size. Then, with five sensors in a single PC, we could achieve a capturing rate of 30 frames per second (obtaining data using all sensors). Furthermore, the proposed skeleton merging algorithm generates results at a speed of 1–2 msec per frame, on average. As a result, the final tracking ran at approximately 28–30 frames per second, allowing real-time tracking of skeleton data. In addition, by connecting multiple devices to each other, all sensors’ time steps were synchronized. Therefore, no additional work for time synchronization was required because the sensor SDK manages the trigger timing between linked sensors.

**Figure 7:**
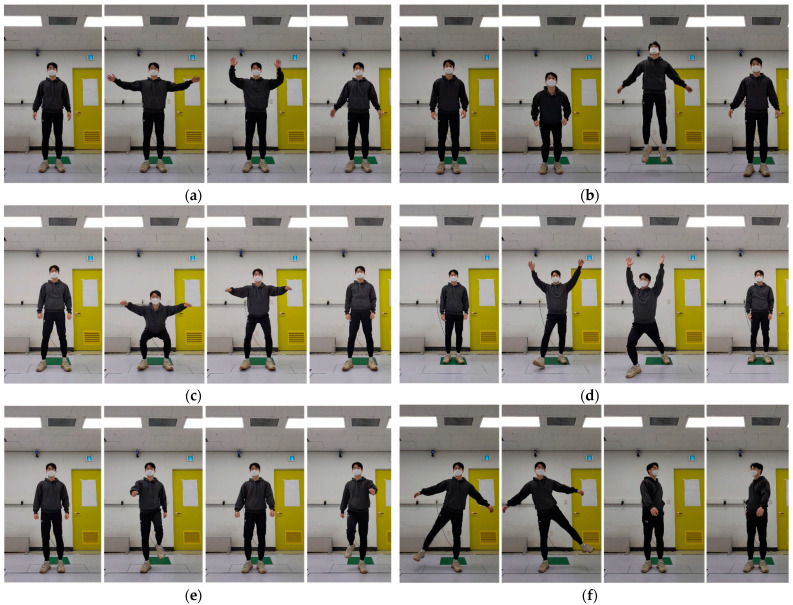
Example of gestures. (**a**) Both hands up and down. (**b**) Jump. (**c**) Squat. (**d**) Lunge. (**e**) Walking. (**f**) Moving body in standing pose (random movement).

We conducted an experiment to evaluate the proposed skeleton tracking algorithm, capturing six gestures performed by six different people using four Azure Kinect sensors. The gestures are both hands up and down, jump, squat, lunge, walking, and moving the body in a standing pose (random movement). The examples of gestures are described in Figure 7. All subjects repeatedly performed each gesture for 1000 frames. Additionally, all gestures were started with a standing pose. In the case of the jump gesture, all subjects jumped naturally with their hands raised. In the case of random movement, all subjects moved their body in standing pose, for example, waving arms, leaning, or crossing arms. Many studies that test the accuracy of skeleton data use motion capture equipment as the ground truth. However, there is an interference problem between the RGB-D sensor and the motion capture system [35,36]. This is caused by the interference of infrared (IR) wavelength between the RGB-D sensor and the IR sensor of the motion capture system. This issue prevents the RGB-D sensor from measuring depth data and causes serious issues when the RGB-D sensor’s body tracker estimates skeleton data. Furthermore, because the retroreflective marker used in the motion capture system reflects IR, the RGB-D sensor cannot extract depth information from the corresponding area, affecting skeleton data recognition. Additionally, the position of each joint in the skeleton data provided by motion capture does not perfectly match the skeleton data of the RGB-D sensor body tracker. As a result, it is not appropriate to use the motion capture system as the ground truth in quantitatively evaluating the proposed algorithm.

Therefore, similar to the evaluation method of [21], we adopted the skeleton data from the best view sensor as the ground truth for the evaluation. The performance of Kinect body tracking is the best when entire body parts could be observed in the depth view of the sensor [14]. Additionally, when the sensor measures the subject from the front view of the subject, the largest number of body parts can be measured [17]. In other words, when the skeleton data are measured from the front view, the result of body tracking SDK could have the best accuracy. Therefore, the skeleton data measured from the front of the subject was adopted as the ground truth. By comparison, we evaluated how different the skeleton data merged by the proposed algorithm was from the ground truth.

**Figure 8:**
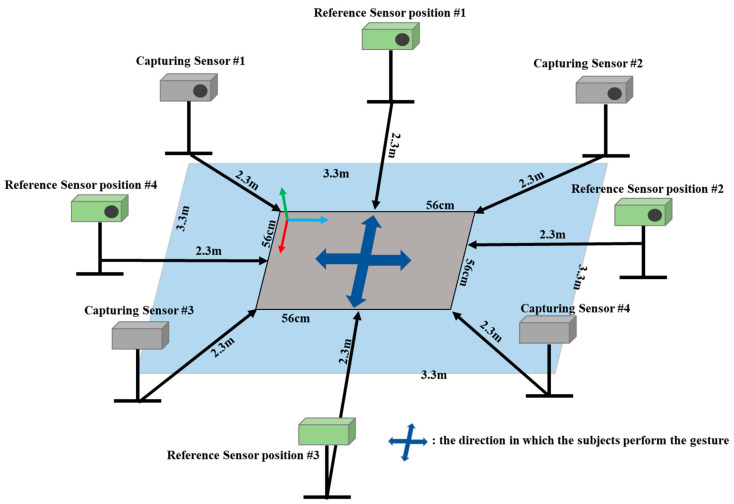
Multiple RGB-D sensors tracking system for evaluation.

For the experiment, we installed four sensors to capture the gestures of subjects and one more sensor was installed additionally for the ground truth. Figure 8 depicts the locations of all capturing sensors with gray color as well as the areas where the subjects performed the gestures. The positions of capturing sensors measuring the skeleton data of the subject were fixed. The subjects performed all gestures for each direction described as the blue arrow-cross in Figure 8. According to directions of the subject, the reference sensor was moved to obtain the reference data that measured the subject from the front (the candidate positions of reference sensor are described in Figure 8 with green color). The best view sensor was capable of capturing all gestures without occlusion.

When all capturing sensors measure the skeleton data of the subject, the self-occlusion problem arises in any direction the subject performs a gesture. We defined the distance between the joint positions of the ground truth and the joint positions of the merging process as an error for the evaluation in millimeters. The standard deviation of the error values is also calculated. Through a designed experiment, the difference between the skeleton data merged by the proposed algorithm and the skeleton data measured from the front was compared. The analysis of the results is described in the following section and the raw data (RMSE, STD) is provided in the Appendix A and Appendix B.

## 3. Experimental Results

### 3.1. Result of Performance Improvement in the Merging Algorithm

**Figure 9:**
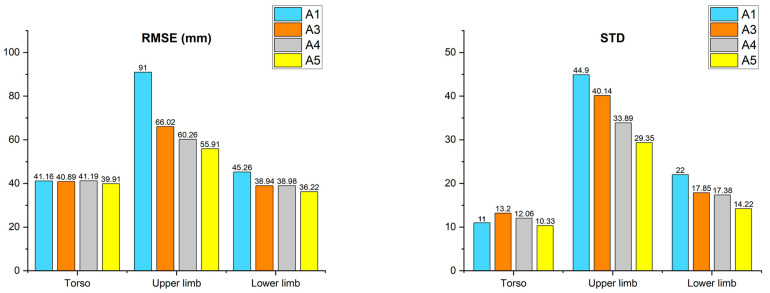
Result of the algorithm experiment (RMSE, STD).

Figure 9 shows the average position error of several algorithms. The first analysis was conducted to prove the performance improvement of the merging algorithm for all gestures performed by all subjects. The comparison group consisted of Just Average (A1), Orientation Resetting and DBSCAN (A3), Orientation Resetting and 1 frame Smoothing DBSCAN (A4), and Orientation Resetting and 1 frame Smoothing DBSCAN with Kalman Filter (A5). Each comparison group represents the elements constituting the proposed merging algorithm. In the case of Orientation Resetting and Average (A2), since there was no significant difference in performance with *A1* in our evaluation data, it was excluded from the comparison group. In other words, there was no misorientation case in evaluation data. However, a misorientation situation was observed when testing the body tracking SDK with a sensor height of 180 cm. Furthermore, since many studies reported the misorientation error of skeleton data measured by Kinect body tracking SDK, the realignment process of the joint’s orientation is determined as essential [21,23,33]. For the result of A5, the average value of the results of setting the DBSCAN searching area to 5, 10, 15, and 20 cm was used. The first experiment’s evaluation is divided into joints corresponding to the torso, upper limb, and lower limb, and the details are as follows.

As a result of A1, the torso joints had an average error (AE) of 41.1 mm, the upper limb joints had an AE of 90.9 mm, and the lower limb joints had an AE of 45.2 mm. Additionally, the standard deviation (STD) was 11.02, 44.89, and 22.0, respectively. In the case of A3, the AE of the torso was 40.8 mm, the upper limb was 66.0 mm, and the lower limb was 38.9 mm, with STD values of 13.1, 40.1, and 17.8, respectively. There was an improvement in joint position accuracy in the joints corresponding to the upper and lower limbs, and the STD of error was reduced in the case of the lower limb, resulting in a smoothing effect. As a result of A4, torso had an AE of 41.1 mm, upper limb 60.2 mm, and lower limb 38.9 mm. STD was 12.0, 33.8, and 17.3, respectively. A4 also showed improvement in performance in the joints corresponding to the upper and lower limbs, also showing a smoothing effect by reducing the STD of error in the joints corresponding to the upper limb. Finally, in the case of A5, the torso had an AE of 39.9 mm, the upper limb had an AE of 55.9 mm, and the lower limb had an AE of 36.2 mm, with the STD of error being 10.3, 29.3, and 14.2, respectively. The performance for positioning accuracy in all joints improved as a result of A5, and the standard deviation of error was also reduced. Consequently, the performance of A5 proposed in this paper had the highest accuracy among all comparison groups. The raw data of the experimental result of algorithm improvement was described in Table A1, Table A2, Table A3 and Table A4 in Appendix A.

### 3.2. Result of Different Searching Areas of DBSCAN

**Figure 10:**
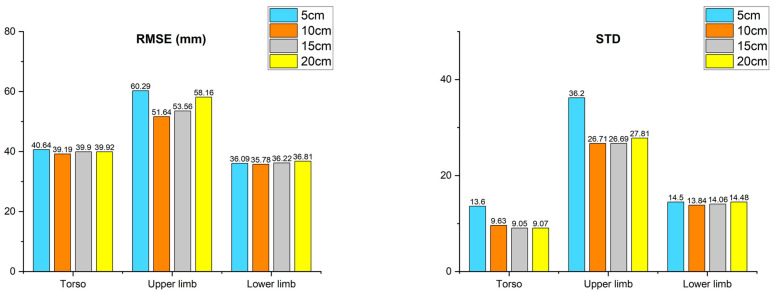
Result of the searching area for DBSCAN.

The second analysis is a comparison of results according to the searching area of DBSCAN used in A3, A4, and A5. Additionally, the comparison group consisted of 5, 10, 15, and 20 cm. As in the above analysis, the results for all gestures are divided into torso, upper limb, and lower limb as shown in Figure 10. As a result of the searching area of 5 cm, the joints of torso had an AE of 40.6 mm, the joints of the upper limb had an AE of 60.2 mm, and the joints of the lower limb had an AE of 36.0 mm. For STD, the results were 13.5, 36.2, and 14.4, respectively. This indicates that the search area is insufficiently large. In particular, in the case of a joint corresponding to a fast-moving limb, a sufficient number of inlier candidates cannot participate in the merging process. Furthermore, the point used for 1 frame smoothing prevents fast-moving inlier candidates from taking part in the merge process. When the searching area was set to 10 cm, the torso had an AE of 39.1 mm, the upper limb had an AE of 51.6 mm, and the lower limb had an AE of 35.7 mm, with the error STD values being 9.6, 26.7, and 13.8, respectively. As a result of setting the searching area to 15 cm, the torso had an AE of 39.9 mm, the upper limb had an AE of 53.5 mm, and the lower limb had an AE of 36.2 mm. Additionally, they had STDs of 9.0, 26.6, and 14.0, respectively. As a result of the searching area of 20 cm, the torso had an AE of 39.9 mm, upper limb 58.1 mm, and lower limb 36.8 mm, with STDs of 9.0, 27.8, and 14.4, respectively. Setting the search area to 15 or 20 cm is too large for noise filtering. As a result, the error increased because points corresponding to noise candidates participated in the merge process without being filtered. Overall, the case of the 10 cm searching area performed the best out of all comparison groups. Furthermore, the standard deviation of the error with searching areas of 15 and 20 cm is less than 10 cm, but there is no statistically significant difference. 

### 3.3. Result According to TNOS

**Figure 11:**
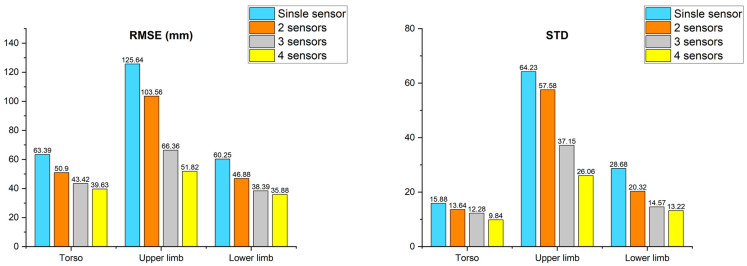
Result of number of sensors.

Finally, a third analysis was performed to evaluate the performance of the proposed algorithm in improving the accuracy of the skeleton data by merging the skeletons obtained from multiple sensors. This analysis also made use of data from the described gestures performed by all subjects. The comparison was carried out by adjusting the TNOS used for merging, and the results are depicted in Figure 11. The TNOS values used in the evaluation are 1, 2, 3, and 4. In the case of a single sensor, the raw data was used instead of the merging algorithm. The comparison components (AE, STD) were calculated as the average value of all combinations with the same size as the TNOS used for merging. In addition, based on the above experimental results, the DBSCAN searching area was fixed to 10 cm that had the best performance.

Regarding the result of a single sensor, the number of combinations was 4, had an AE of 63.3 mm for the joints corresponding to the torso, 125.6 mm for the joints belonging to the upper limb, and 60.2 mm for the lower limb. The STD values of error were 15.8, 64.2, and 28.6, respectively. The number of combinations in the case of using two sensors for merging was 6, and the torso had an AE of 50.9 mm, the upper limb had an AE of 103.5 mm, and the lower limb had an AE of 46.8 mm. Additionally, the standard deviations of error were 13.6, 57.5, and 20.3, respectively. The number of combinations for the use of three sensors was 4, and the AE of torso joints was 43.4 mm, upper limb 66.3 mm, and lower limb 38.3 mm. The standard deviations of error were 12.2, 37.1, and 14.5, respectively. Lastly, in the case of four sensors with a single combination, the AE of torso joints was 39.6 mm, the upper limb 51.8 mm, and the lower limb 35.8 mm. Additionally, the STD values of error were 9.8, 26.0, and 13.2, respectively. Consequently, the accuracy of merged skeleton data increased according to the increase in the TNOS used for merging. The result of TONS experiment was described in Table A5 and Table A6 in Appendix B.

## 4. Discussion

In this study, we proposed the markerless skeleton tracking algorithm to track skeleton data accurately. The main strategy of the algorithm is filtering the noise candidate joints that occurred due to the self-occlusion problem in the merging process. The proposed algorithm was evaluated by comparing the ground truth obtained with the best view sensor that measures the subject from the front. The detailed analysis for this experimental result is as follows: The analysis was conducted based on the results of the searching area of 10 cm with the best performance. Among the joints corresponding to the torso (pelvis, spine naval, neck, left hip, right hip, left shoulder, right shoulder, and head joints) they could be measured in all gestures because of the low installation height of all sensors. Furthermore, because the amount of change in the positions of the torso joints was not large while the subjects performed all gestures, the proposed algorithm outperformed A1 by less than 7 mm.

In the case of the upper limb joints, the proposed algorithm showed at least 15 mm better results than A1 in all gestures. Among them, the squat gesture improved performance the most. Furthermore, when compared to the results of the elbow joint, the performance of the wrist joint improved by an average of 10 mm or more. In comparison to A1, A3 improved by 20.1 mm, A4 by 23.4 mm, and A5 by 27.0 mm as a result of the elbow joint in all gestures. In the case of the squat gesture, the A3 improved by 34.3 mm, the A4 by 39.3 mm, and the A5 by 42.7 mm for elbow joint. Moreover, in the case of the wrist joint, there was an improvement in the performance of 29.8 mm for A3, 45.4 mm for A4, and 51.6 mm for A5 compared with A1 for all gestures. Additionally, the same as in the case of the elbow, it showed the maximum performance in the squat gesture, and there were performance improvements of 44.0, 80.0, and 83.9 mm for each algorithm. The subjects frequently raised their upper limb toward the sensor during the squatting gesture, resulting in self-occlusion problems at the elbow and wrist joints.

In the case of the joints corresponding to the lower limb, the difference in improvement of performance between the knee and ankle joints is not large, within 7 mm except for the lunge gesture. This is because, due to the characteristics of all gestures, the effect of occlusion affecting the lower limb was not large. Thus, we described the result of the lunge gesture below. In the results of the knee joint, the coordinate accuracy of the joint corresponding to the lower limb was improved by 25.5 mm in A3, 27.7 mm in A4, and 27.6 mm in A5 compared with A1, respectively. Additionally, in the case of the ankle joint, the improvement of A3 was 28.4 mm, A4 32.2 mm, A5 34.5 mm, respectively. Consequently, while there was a large error in the result of A1 in the merged skeleton, the skeletal data composed of the precise joint position could be obtained using the algorithm proposed in this paper.

Our results are comparable to the performance in existing studies related to tracking accurate skeleton data by merging multiple inaccurate skeleton data. Existing skeleton merging algorithms were mainly developed on the basis of Kinect V2. In addition, the superiority of the algorithm was evaluated compared to the performance of a single Kinect. In [22], the authors reported an error of 87 mm for the entire joint in the T-pose and walking around gesture. For the experiment, eight Kinect v2 sensors were used and a marker-based motion capture system was used as the ground truth. They mentioned that the position of the skeleton recognized using the motion capture device and the skeleton of the Kinect SDK did not match perfectly, so there was a problem in accurate comparison. To overcome this, the position of the marker closest to the skeleton joint among the markers attached to the body of the target was adopted as the ground truth, and there was an average distance difference of 100 mm between the joint of skeleton data and marker position. In [23], a merged skeleton was obtained using seven Kinect V2 sensors, and the skeleton data measured in the best view was adopted as the ground truth in the same way as in this study. They reported an average error of 80.3 mm in the experiments on standing, rotating, walking, roaming, and free motion gestures performed by seven subjects. The best view sensor was automatically selected among the capturing sensors using the factor used in the PJA algorithm proposed in the paper. In [24], the authors merged skeletons obtained from five Kinect V2 sensors into one skeleton. They adopted a marker-based motion capture system as the ground truth and evaluated the performance of the proposed merged algorithm in terms of the gestures of walking, spinning, sitting, running, kicking, punching, and crossing of limbs. As a result, the average errors for all joints were 97.1, 91.2, and 69.5 mm for single Kinect (centre-Kinect), simply average, and the proposed merging method, respectively. They also reported that motion capture skeleton and Kinect skeleton did not match perfectly, as in [22], and there was an average difference of 55 mm between the skeleton of Kinect and motion capture system. In [26], the authors obtained a merged skeleton using three Kinect V2 sensors. They evaluated the performance of the proposed merging algorithm using a specific training protocol and non-standing posture, including standing, bending, squat, lying, crossing arms or legs. They also adopted the skeleton obtained from the marker-based motion capture system as the ground truth. As a result, they reported that the accuracy of the merged skeleton was improved by 15.7% compared to the single Kinect, and the average error was measured to be less than 55 mm for all joints.

As with the results in this study, most of the studies have reported that the performance improvement in the joints belonging to the limbs is more pronounced compared to the torso joints. Ref. [26] also reported that the joint belonging to the limbs had a higher performance improvement than the torso. In [25], the authors obtained merged skeleton data of 20 people walking on the treadmill using five Kinect V2 sensors. They evaluated the performance of the merging algorithm using the STD for the difference between the pre-measured bone length of the subjects and that of the merged skeleton. As a result, the STD of the torso was 5.9, 12.0 for the upper limb and 21.8 for the lower limb. In [19], the authors proposed a skeleton merging algorithm for each Kinect V2 and Azure Kinect skeleton. Three of each sensor types were used, and a marker-based motion capture system was adopted as the ground truth. They evaluated the proposed algorithm for running, kicking, punching, crossing arms, crossing legs, crossing arms and legs, bowing from the waist, sitting on the chair, spinning, and walking around. As a result, Kinect V2 reported an error of 46.2 mm for the torso joints, 105 mm for the upper limb, and 135.5 mm for the lower limb. According to the Azure Kinect result, an error of 31 mm for the torso, 59.5 mm for the upper limb, and 121.5 for the lower limb was measured. As mentioned before, the interference problem and IR-reflective marker issue were reported also. For this issue, controlling the trigger between the motion capture system and Azure Kinect was necessary. Additionally, the miniature markers of 2.5 mm in diameter were used to avoid the problem that depth information is not measured by the markers. However, despite these attempts, the problem that occurs when the motion capture system and Azure Kinect are running at the same time cannot be completely eliminated. In [21], the authors proposed a skeleton merging algorithm using four Kinect V2 sensors. Similar to this study, the skeleton measured using the manually selected best view sensor was adopted as the ground truth. The authors collected validation data for walking, walking and spinning, walking while moving arms, walking and bending down, spin arms, and jumping jack gestures performed by six subjects (three for training; three for testing). The performance of the proposed algorithm was evaluated by comparing with the results of a single sensor. As a result, the error for the single sensor was 128.6 mm in the torso, 187.2 mm in the upper limb, and 157.3 mm in the lower limb. The error for the merged skeleton was 86.0 mm in the torso, 94.5 mm in the upper limb, and 97.5 mm in the lower limb.

As a result of analyzing the performance improvement of the components constituting the algorithm, the merged skeleton using A5 (Orientation Resetting and 1 frame Smoothing DBSCAN with Kalman Filter) was the most accurate. Furthermore, the highest accuracy was obtained when 10 cm was applied to the DBSCAN searching area. Similar to the results of other research, the proposed algorithm improved the accuracy of the merged skeleton compared to the single sensor, and the accuracy improvement for the joints belonging to the limbs was greater than the joints belonging to the torso. In addition, our result was comparable or greater to the performances in existing studies about development of skeleton merging algorithms. Especially, the error of the proposed algorithm was relatively low compared to [21,23], which used the best view evaluation method as in this study. In addition, the accuracy of the merged skeleton increased as TNOS increased. However, the evaluation method performed to evaluate the performance of the proposed algorithm only evaluates the difference between the merged skeleton and the skeleton captured using the best view. Therefore, in order to evaluate the performance of a wider range, it is necessary to compare it with an interference-free measuring device that can measure actual human behavior, such as motion capture using IMU.

Consequently, the proposed system can track the skeleton as accurately as a skeleton measured in front of the subject. The proposed system can be utilized in the field of behavioral monitoring research targeting human tracking. Furthermore, it can be used for a variety of interactive content such as games or education. The proposed algorithm, on the other hand, is intended to apply to a single person. In this study, we focus on applying the algorithm to only a single person. Therefore, extending the study is needed to apply the proposed algorithm for tracking multiple people. For the next study, we have a plan to implement a game-like interaction with a large number of participants. A study on the standards of gestures (actual motion shape and speed) that can be actually applied by using the skeleton data will be conducted to define the possible interaction. Additionally, discussion of the proper installation of sensors to track multiple people will also be covered.

## 5. Conclusions

The goal of this paper was development of the markerless skeleton tracking system using multiple RGB-D sensors. We proposed the algorithm to merge multiple skeleton data, which includes an error in the position of joints due to self-occlusion problems, into accurate single skeleton data. The main issue with this approach was determining how to filter the noise candidates reported by the multiple RGB-D sensors during the merging process. To address this issue, we used a clustering algorithm called DBSCAN. We proposed additional tricks to increase the weight of inlier candidates participating in the merging process. For the evaluation of the proposed algorithm, we conducted the experiment capturing the six gestures performed by six subjects using four capturing RGB-D sensors and a single best view sensor for obtaining ground truth. As a result of the analysis, the proposed algorithm showed the most accurate performance. Additionally, the proposed method showed relatively lower errors than other related studies. Furthermore, the result of the 10 cm searching area of DBSCAN showed the highest accuracy. Consequently, using the algorithm proposed in this study, it was possible to acquire skeleton data as accurate as the skeleton data measured from the front of the subject.

## Appendix A

**Table A1:** RMSE result of the algorithm development experiment for gestures 1, 2, and 3.

**G1**	**RMSE**	**A1**	**A3_5**	**A3_10**	**A3_15**	**A3_20**	**A4_5**	**A4_10**	**A4_15**	**A4_20**	**A5_5**	**A5_10**	**A5_15**	**A5_20**
	**Pelvis**	30.40	31.09	31.09	31.09	31.09	32.27	30.40	30.40	30.40	32.05	30.28	30.28	30.28
	**Spine Naval**	45.32	47.72	47.72	47.72	47.72	48.08	45.32	45.32	45.32	47.91	45.15	45.16	45.16
	**Neck**	39.13	35.34	35.34	35.34	35.34	32.66	39.16	39.14	39.14	30.84	38.69	38.68	38.68
	**L Shoulder**	39.11	33.58	33.58	33.58	33.58	31.27	36.55	39.11	39.11	30.10	35.48	38.48	38.48
	**L Elbow**	53.92	40.58	40.58	40.58	40.58	39.46	39.15	40.94	43.75	37.09	37.84	39.66	42.59
	**L Wrist**	80.86	65.02	65.02	65.02	65.02	65.12	59.06	57.98	59.96	56.85	54.25	55.95	57.83
	**R Shoulder**	33.82	36.67	36.67	36.67	36.67	38.44	32.88	33.90	33.86	37.35	31.80	33.11	33.05
	**R Elbow**	57.42	41.22	41.22	41.22	41.22	40.35	34.60	35.38	39.37	36.49	33.29	33.53	37.51
	**R Wrist**	102.76	72.64	72.64	72.64	72.64	74.84	59.57	58.64	57.83	68.68	54.33	56.34	56.33
	**L Hip**	33.17	36.36	36.36	36.36	36.36	38.89	33.17	33.17	33.17	38.43	33.00	33.00	33.00
	**L Knee**	24.80	24.13	24.13	24.13	24.13	24.16	24.77	24.79	24.80	23.74	24.34	24.37	24.38
	**L Ankle**	31.51	31.67	31.67	31.67	31.67	32.18	31.02	31.30	31.51	31.49	30.38	30.61	30.84
	**R Hip**	29.36	31.79	31.79	31.79	31.79	31.57	29.35	29.36	29.36	31.27	29.17	29.17	29.17
	**R Knee**	22.83	22.91	22.91	22.91	22.91	23.16	22.82	22.82	22.83	22.85	22.51	22.51	22.51
	**R Ankle**	29.44	28.26	28.26	28.26	28.26	28.53	28.77	28.93	29.48	27.88	28.18	28.16	28.86
	**Head**	38.32	34.42	34.42	34.42	34.42	32.10	38.28	38.32	38.32	30.49	37.53	37.62	37.61
**G2**	**Pelvis**	35.32	35.87	35.87	35.87	35.87	36.74	35.29	35.32	35.32	32.93	32.71	32.75	32.75
	**Spine Naval**	48.80	46.31	46.31	46.31	46.31	56.12	48.51	48.80	48.80	50.61	46.61	46.92	46.92
	**Neck**	50.75	49.68	49.68	49.68	49.68	51.88	50.33	50.76	50.76	47.64	48.06	48.52	48.52
	**L Shoulder**	49.58	45.94	45.94	45.94	45.94	47.87	44.89	49.47	49.57	42.72	40.78	46.22	46.34
	**L Elbow**	73.62	56.68	56.68	56.68	56.68	57.10	53.40	56.65	64.31	47.99	45.59	50.11	59.17
	**L Wrist**	114.99	91.85	91.85	91.85	91.85	91.57	82.09	80.16	85.49	83.13	69.29	69.07	75.93
	**R Shoulder**	46.74	49.71	49.71	49.71	49.71	54.02	45.10	46.73	46.74	48.23	41.07	43.33	43.33
	**R Elbow**	72.43	60.63	60.63	60.63	60.63	67.36	59.09	55.69	60.33	56.17	48.71	49.07	54.37
	**R Wrist**	121.59	95.22	95.22	95.22	95.22	98.57	86.13	81.28	78.73	95.07	73.30	70.18	69.41
	**L Hip**	37.93	37.87	37.87	37.87	37.87	39.95	37.81	37.93	37.93	34.94	34.92	35.08	35.08
	**L Knee**	44.43	44.87	44.87	44.87	44.87	45.94	44.31	44.37	44.42	40.24	39.32	39.37	39.41
	**L Ankle**	43.33	43.29	43.29	43.29	43.29	43.67	42.94	42.99	43.12	38.21	37.83	37.89	37.99
	**R Hip**	35.16	35.91	35.91	35.91	35.91	37.77	35.09	35.17	35.16	33.46	32.02	32.18	32.17
	**R Knee**	40.94	41.43	41.43	41.43	41.43	42.74	40.82	40.82	40.93	36.45	35.41	35.43	35.49
	**R Ankle**	40.84	40.18	40.18	40.18	40.18	40.44	39.85	39.96	40.10	34.67	34.47	34.59	34.68
	**Head**	52.50	52.72	52.72	52.72	52.72	55.28	52.09	52.48	52.50	49.92	49.43	49.91	49.94
**G3**	**Pelvis**	39.07	39.38	39.38	39.38	39.38	39.62	39.07	39.07	39.07	38.39	37.86	37.86	37.86
	**Spine Naval**	49.01	49.06	49.06	49.06	49.06	50.01	49.00	49.01	49.01	49.76	48.22	48.23	48.23
	**Neck**	45.79	47.42	47.42	47.42	47.42	48.69	45.73	45.75	45.79	45.16	44.71	44.73	44.77
	**L Shoulder**	44.68	43.02	43.02	43.02	43.02	42.65	42.37	44.65	44.66	41.43	40.33	43.07	43.08
	**L Elbow**	84.43	47.26	47.26	47.26	47.26	44.03	43.43	50.82	58.81	41.34	40.99	48.08	56.36
	**L Wrist**	145.80	104.66	104.66	104.66	104.66	90.87	70.54	75.03	82.32	92.94	66.62	72.43	79.77
	**R Shoulder**	36.63	42.41	42.41	42.41	42.41	45.17	35.53	36.55	36.62	42.95	33.42	34.72	34.77
	**R Elbow**	78.15	46.72	46.72	46.72	46.72	43.49	40.45	44.93	51.04	38.73	36.15	39.05	45.78
	**R Wrist**	145.46	98.59	98.59	98.59	98.59	97.68	60.66	62.45	69.23	104.87	56.75	58.79	64.79
	**L Hip**	40.82	41.27	41.27	41.27	41.27	42.92	40.82	40.82	40.82	41.73	39.52	39.52	39.52
	**L Knee**	44.14	39.60	39.60	39.60	39.60	39.58	39.91	40.79	41.52	38.07	37.96	38.76	39.32
	**L Ankle**	45.07	41.78	41.78	41.78	41.78	42.07	41.21	41.55	42.22	39.05	38.22	38.41	38.78
	**R Hip**	38.65	38.92	38.92	38.92	38.92	39.70	38.65	38.65	38.65	38.33	37.25	37.25	37.25
	**R Knee**	31.63	31.27	31.27	31.27	31.27	31.69	31.54	31.55	31.59	29.35	29.14	29.15	29.18
	**R Ankle**	33.99	33.07	33.07	33.07	33.07	33.20	33.57	33.70	33.80	29.89	30.36	30.45	30.53
	**Head**	45.89	49.01	49.01	49.01	49.01	50.92	45.82	45.81	45.88	47.31	44.59	44.58	44.64

**Table A2:** RMSE result of the algorithm development experiment for gestures 4, 5, and 6.

**G4**	**RMSE**	**A1**	**A3_5**	**A3_10**	**A3_15**	**A3_20**	**A4_5**	**A4_10**	**A4_15**	**A4_20**	**A5_5**	**A5_10**	**A5_15**	**A5_20**
	**Pelvis**	33.94	35.27	35.27	35.27	35.27	37.49	34.01	33.94	33.94	36.93	33.46	33.37	33.37
	**Spine Naval**	46.02	47.81	47.81	47.81	47.81	48.82	45.66	46.02	46.02	49.95	45.09	45.47	45.47
	**Neck**	41.99	38.10	38.10	38.10	38.10	39.04	41.75	41.99	41.99	39.78	40.72	40.98	40.98
	**L Shoulder**	44.50	39.82	39.82	39.82	39.82	42.27	39.69	43.84	44.29	41.53	38.12	42.33	42.75
	**L Elbow**	74.18	51.85	51.85	51.85	51.85	50.64	45.99	48.18	54.01	47.88	43.99	47.03	53.00
	**L Wrist**	109.44	81.04	81.04	81.04	81.04	75.88	65.34	63.65	67.16	69.52	59.35	61.87	66.62
	**R Shoulder**	38.45	39.96	39.96	39.96	39.96	43.66	37.29	38.08	38.27	42.65	35.48	36.32	36.50
	**R Elbow**	64.24	48.17	48.17	48.17	48.17	49.82	43.17	44.13	47.34	43.47	40.92	41.26	44.08
	**R Wrist**	111.15	78.47	78.47	78.47	78.47	77.60	62.44	60.09	63.11	76.96	56.12	56.97	60.64
	**L Hip**	37.48	38.75	38.75	38.75	38.75	41.67	36.92	37.47	37.48	40.56	36.18	36.69	36.69
	**L Knee**	66.81	42.99	42.99	42.99	42.99	43.34	40.91	42.25	44.54	42.65	40.54	41.50	43.36
	**L Ankle**	76.47	50.94	50.94	50.94	50.94	48.78	47.76	49.60	51.21	45.33	45.32	47.26	48.59
	**R Hip**	33.06	34.37	34.37	34.37	34.37	37.09	33.49	33.05	33.06	36.62	32.82	32.28	32.28
	**R Knee**	67.24	40.02	40.02	40.02	40.02	40.73	37.73	38.75	40.96	39.72	38.23	38.71	40.64
	**R Ankle**	85.21	53.90	53.90	53.90	53.90	51.48	49.38	50.65	52.72	46.93	47.35	49.19	50.37
	**Head**	42.40	38.29	38.29	38.29	38.29	39.30	42.02	42.39	42.40	39.23	40.71	41.07	41.08
**G5**	**Pelvis**	32.73	34.34	34.34	34.34	34.34	34.73	32.73	32.73	32.73	32.70	31.84	31.84	31.84
	**Spine Naval**	45.16	44.28	44.28	44.28	44.28	45.60	44.99	45.16	45.16	49.05	44.61	44.78	44.78
	**Neck**	51.96	49.37	49.37	49.37	49.37	49.56	51.96	51.96	51.96	49.23	51.44	51.39	51.39
	**L Shoulder**	48.14	44.49	44.49	44.49	44.49	43.55	44.00	48.15	48.14	44.16	42.96	46.68	46.67
	**L Elbow**	80.28	52.75	52.75	52.75	52.75	53.27	54.56	61.44	70.22	48.24	54.03	57.10	66.28
	**L Wrist**	121.88	88.16	88.16	88.16	88.16	85.41	76.12	76.53	82.84	80.97	74.79	76.55	79.22
	**R Shoulder**	42.72	49.08	49.08	49.08	49.08	51.77	43.00	42.72	42.72	51.69	40.97	41.13	41.13
	**R Elbow**	65.98	46.88	46.88	46.88	46.88	48.98	44.23	46.66	52.32	44.32	41.33	41.96	47.58
	**R Wrist**	120.28	88.29	88.29	88.29	88.29	82.33	71.00	72.44	76.62	77.43	68.48	70.13	72.80
	**L Hip**	35.70	37.43	37.43	37.43	37.43	39.34	35.71	35.70	35.70	37.95	34.47	34.47	34.47
	**L Knee**	54.21	46.71	46.71	46.71	46.71	47.37	46.60	47.43	48.25	42.63	42.49	43.23	43.79
	**L Ankle**	57.21	52.51	52.51	52.51	52.51	52.52	51.53	51.87	53.02	47.55	46.92	47.15	48.12
	**R Hip**	33.16	31.73	31.73	31.73	31.73	31.16	32.96	33.16	33.16	28.79	31.63	31.86	31.86
	**R Knee**	54.25	45.22	45.22	45.22	45.22	47.25	44.90	45.94	47.42	42.07	41.61	42.44	43.65
	**R Ankle**	52.10	46.15	46.15	46.15	46.15	46.15	44.95	45.95	48.50	41.09	40.82	41.64	43.96
	**Head**	55.34	53.60	53.60	53.60	53.60	54.85	56.19	55.34	55.34	56.60	55.49	54.59	54.59
**G6**	**Pelvis**	34.99	36.77	36.77	36.77	36.77	37.17	34.99	34.99	34.99	37.48	34.42	34.42	34.42
	**Spine Naval**	46.54	47.07	47.07	47.07	47.07	45.30	46.41	46.54	46.54	44.77	46.03	46.18	46.18
	**Neck**	42.71	36.52	36.52	36.52	36.52	33.19	42.67	42.71	42.71	34.14	42.05	42.11	42.10
	**L Shoulder**	44.50	39.47	39.47	39.47	39.47	38.80	40.19	44.37	44.39	38.36	38.32	42.94	42.97
	**L Elbow**	61.36	43.70	43.70	43.70	43.70	43.34	42.28	46.05	51.66	39.49	38.58	42.15	48.13
	**L Wrist**	90.33	67.82	67.82	67.82	67.82	65.07	59.62	61.46	65.48	57.21	54.08	55.82	59.87
	**R Shoulder**	41.48	42.54	42.54	42.54	42.54	44.29	40.50	41.41	41.40	42.70	38.70	39.86	39.86
	**R Elbow**	59.40	47.07	47.07	47.07	47.07	45.62	43.32	44.39	48.44	41.24	39.57	40.15	44.02
	**R Wrist**	93.98	69.09	69.09	69.09	69.09	66.93	60.72	57.65	59.33	60.77	50.91	52.09	53.72
	**L Hip**	38.45	39.65	39.65	39.65	39.65	41.16	37.55	38.45	38.45	41.14	36.65	37.60	37.60
	**L Knee**	33.51	32.00	32.00	32.00	32.00	32.18	33.02	33.19	33.39	30.63	31.26	31.41	31.58
	**L Ankle**	38.39	36.27	36.27	36.27	36.27	36.42	36.84	37.36	37.55	33.90	34.50	35.01	35.21
	**R Hip**	35.53	36.05	36.05	36.05	36.05	36.23	35.43	35.55	35.54	35.80	34.56	34.71	34.69
	**R Knee**	30.37	30.35	30.35	30.35	30.35	30.67	29.91	29.97	30.08	29.13	28.45	28.49	28.57
	**R Ankle**	37.43	35.09	35.09	35.09	35.09	34.99	35.50	35.82	36.06	32.58	33.13	33.44	33.60
	**Head**	42.75	36.68	36.68	36.68	36.68	35.26	42.73	42.75	42.74	35.15	41.88	41.90	41.90

**Table A3:** STD result of the algorithm development experiment for gestures 1, 2, and 3.

**G1**	**STD**	**A1**	**A3_5**	**A3_10**	**A3_15**	**A3_20**	**A4_5**	**A4_10**	**A4_15**	**A4_20**	**A5_5**	**A5_10**	**A5_15**	**A5_20**
	**Pelvis**	3.45	4.45	4.45	4.45	4.45	4.43	3.45	3.45	3.45	4.08	3.17	3.17	3.18
	**Spine Naval**	6.10	11.24	11.24	11.24	11.24	13.81	6.10	6.09	6.09	12.52	5.75	5.74	5.74
	**Neck**	9.06	12.76	12.76	12.76	12.76	13.84	9.07	6.09	9.07	12.23	8.43	8.44	8.43
	**L Shoulder**	7.78	10.71	10.71	10.71	10.71	10.77	10.36	7.77	7.77	9.57	9.10	6.93	6.94
	**L Elbow**	23.15	18.92	18.92	18.92	18.92	17.38	14.38	14.87	15.70	14.72	12.97	13.39	14.20
	**L Wrist**	42.49	37.60	37.60	37.60	37.60	36.66	30.24	28.15	28.94	30.69	26.18	26.49	27.01
	**R Shoulder**	8.89	12.85	12.85	12.85	12.85	13.65	8.93	8.94	8.92	12.34	7.85	8.12	8.10
	**R Elbow**	28.68	22.19	22.19	22.19	22.19	20.05	14.53	15.45	18.10	16.44	12.98	13.88	16.60
	**R Wrist**	52.66	45.37	45.37	45.37	45.37	48.06	33.30	32.03	29.95	44.71	28.59	28.88	27.70
	**L Hip**	3.79	4.85	4.85	4.85	4.85	5.26	3.79	3.79	3.79	4.77	3.40	3.40	3.40
	**L Knee**	5.36	5.13	5.13	5.13	5.13	5.15	5.32	5.35	5.36	4.46	4.60	4.63	4.64
	**L Ankle**	7.03	6.85	6.85	6.85	6.85	6.92	6.76	6.93	7.03	5.91	5.73	5.84	5.93
	**R Hip**	3.98	5.69	5.69	5.69	5.69	6.82	3.99	3.98	3.98	6.12	3.59	3.57	3.57
	**R Knee**	4.85	4.91	4.91	4.91	4.91	4.96	4.82	4.83	4.85	4.37	4.24	4.24	4.24
	**R Ankle**	7.93	8.13	8.13	8.13	8.13	8.05	7.85	8.00	7.97	7.03	6.84	6.92	6.86
	**Head**	10.70	13.16	13.16	13.16	13.16	13.66	10.84	10.71	10.70	12.37	10.11	9.98	9.98
**G2**	**Pelvis**	12.79	14.41	14.41	14.41	14.41	15.54	12.78	12.79	12.79	11.44	9.94	9.97	9.97
	**Spine Naval**	10.86	15.24	15.24	15.24	15.24	26.71	10.58	10.85	10.86	22.01	8.03	8.30	8.31
	**Neck**	12.80	16.46	16.46	16.46	16.46	23.79	12.59	12.79	12.79	19.39	9.81	10.06	10.06
	**L Shoulder**	14.74	18.44	18.44	18.44	18.44	21.35	18.79	14.68	14.74	15.22	14.14	10.45	10.55
	**L Elbow**	33.16	33.61	33.61	33.61	33.61	33.92	30.21	30.36	29.39	25.80	22.49	23.50	23.88
	**L Wrist**	51.15	54.68	54.68	54.68	54.68	57.65	48.94	45.46	46.73	51.72	37.40	34.74	37.18
	**R Shoulder**	16.48	17.89	17.89	17.89	17.89	21.06	17.61	16.48	16.49	15.42	13.31	12.50	12.51
	**R Elbow**	36.55	38.39	38.39	38.39	38.39	45.24	37.63	33.72	32.68	35.36	27.56	27.52	27.06
	**R Wrist**	55.10	60.51	60.51	60.51	60.51	67.01	56.81	51.07	46.69	66.02	45.89	41.36	37.84
	**L Hip**	13.04	15.37	15.37	15.37	15.37	17.08	13.07	13.04	13.04	12.08	9.78	9.78	9.77
	**L Knee**	20.11	21.28	21.28	21.28	21.28	22.43	20.02	20.05	20.10	17.05	15.43	15.45	15.50
	**L Ankle**	20.19	20.24	20.24	20.24	20.24	20.52	19.88	19.93	19.98	15.36	14.97	15.05	15.03
	**R Hip**	14.25	15.57	15.57	15.57	15.57	16.40	14.32	14.26	14.25	11.90	10.93	10.89	10.88
	**R Knee**	20.85	21.39	21.39	21.39	21.39	22.62	20.77	20.76	20.83	16.61	15.68	15.69	15.74
	**R Ankle**	21.28	20.80	20.80	20.80	20.80	20.89	20.31	20.32	20.43	15.44	15.17	15.182	15.27
	**Head**	14.57	17.65	17.65	17.65	17.65	23.13	14.56	14.55	14.57	18.81	11.57	11.67	11.71
**G3**	**Pelvis**	11.40	12.23	12.23	12.23	12.23	12.48	11.40	11.40	11.40	10.75	9.76	9.76	9.76
	**Spine Naval**	10.46	13.42	13.42	13.42	13.42	17.93	10.46	10.46	10.46	18.11	9.22	9.23	9.23
	**Neck**	12.20	13.94	13.94	13.94	13.94	16.91	12.12	12.15	12.20	13.85	11.02	11.05	11.10
	**L Shoulder**	12.91	14.50	14.50	14.50	14.50	15.01	15.30	12.88	12.90	12.65	12.62	10.46	10.49
	**L Elbow**	41.10	26.47	26.47	26.47	26.47	22.51	21.61	25.21	28.33	18.34	17.56	20.72	23.43
	**L Wrist**	71.55	65.38	65.38	65.38	65.38	57.84	37.17	37.79	39.90	58.31	31.50	33.64	35.58
	**R Shoulder**	12.59	15.19	15.19	15.19	15.19	15.71	12.35	12.50	12.59	13.86	10.00	10.37	10.44
	**R Elbow**	41.46	29.33	29.33	29.33	29.33	24.94	21.66	25.70	29.41	19.26	16.19	19.14	23.20
	**R Wrist**	70.93	66.74	66.74	66.74	66.74	72.51	35.83	35.12	39.94	78.49	31.37	30.60	34.25
	**L Hip**	11.36	12.24	12.24	12.24	12.24	12.49	11.35	11.36	11.36	10.80	9.45	9.46	9.46
	**L Knee**	19.77	16.06	16.06	16.06	16.06	15.74	15.39	16.18	16.94	13.13	12.54	13.30	13.87
	**L Ankle**	20.15	17.00	17.00	17.00	17.00	17.22	16.70	16.90	17.48	13.47	13.00	13.08	13.27
	**R Hip**	12.63	13.83	13.83	13.83	13.83	14.03	12.63	12.63	12.63	12.06	10.73	10.73	10.73
	**R Knee**	14.64	14.69	14.69	14.69	14.69	15.14	14.59	14.59	14.61	12.45	11.92	11.91	11.91
	**R Ankle**	16.19	15.50	15.50	15.50	15.50	15.68	15.82	15.88	15.95	11.88	12.13	12.14	12.19
	**Head**	13.53	15.43	15.43	15.43	15.43	17.73	13.43	13.44	13.52	15.03	12.29	12.30	12.38

**Table A4:** STD result of the algorithm development experiment for gestures 4, 5, and 6.

**G4**	**STD**	**A1**	**A3_5**	**A3_10**	**A3_15**	**A3_20**	**A4_5**	**A4_10**	**A4_15**	**A4_20**	**A5_5**	**A5_10**	**A5_15**	**A5_20**
	**Pelvis**	10.41	12.55	12.55	12.55	12.55	14.55	10.55	10.41	10.41	13.92	9.97	9.81	9.81
	**Spine Naval**	10.04	16.52	16.52	16.52	16.52	22.98	10.43	10.04	10.04	23.54	9.85	9.43	9.43
	**Neck**	12.45	15.17	15.17	15.17	15.17	18.27	12.55	12.45	12.45	18.03	11.52	11.39	11.39
	**L Shoulder**	14.45	17.30	17.30	17.30	17.30	20.23	15.97	13.80	14.20	19.37	14.07	12.00	12.34
	**L Elbow**	38.09	29.80	29.80	29.80	29.80	27.89	22.40	23.23	26.32	25.72	18.72	20.71	23.97
	**L Wrist**	61.16	53.22	53.22	53.22	53.22	50.01	39.65	36.59	38.05	45.13	33.78	34.28	36.44
	**R Shoulder**	14.44	16.36	16.36	16.36	16.36	18.07	14.46	14.10	14.28	17.16	12.82	12.32	12.53
	**R Elbow**	34.64	28.33	28.33	28.33	28.33	28.87	23.11	23.76	25.23	22.24	19.41	20.11	21.41
	**R Wrist**	61.44	52.08	52.08	52.08	52.08	54.26	38.87	35.24	37.12	55.49	32.81	31.15	33.61
	**L Hip**	11.21	13.86	13.86	13.86	13.86	15.97	11.42	11.21	11.21	15.62	10.52	10.27	10.27
	**L Knee**	35.51	19.96	19.96	19.96	19.96	20.00	17.07	17.75	19.47	19.28	16.60	16.89	18.08
	**L Ankle**	40.77	25.19	25.19	25.19	25.19	23.09	21.28	22.12	22.88	19.78	19.01	20.05	20.42
	**R Hip**	11.50	13.28	13.28	13.28	13.28	15.01	11.98	11.50	11.50	14.13	11.34	10.67	10.67
	**R Knee**	38.04	18.94	18.94	18.94	18.94	19.06	16.27	16.87	18.55	17.56	16.01	16.25	17.72
	**R Ankle**	44.79	28.22	28.22	28.22	28.22	26.13	23.20	23.64	25.33	21.64	21.09	22.05	22.83
	**Head**	13.98	15.60	15.60	15.60	15.60	17.87	13.98	13.98	13.98	17.46	12.78	12.74	12.74
**G5**	**Pelvis**	8.02	9.84	9.84	9.84	9.84	10.11	8.02	8.02	8.02	7.97	6.88	6.88	6.88
	**Spine Naval**	6.00	10.46	10.46	10.46	10.46	16.43	6.15	6.00	6.00	17.35	5.14	5.04	5.04
	**Neck**	8.95	11.83	11.83	11.83	11.83	16.22	9.31	8.95	8.95	16.61	8.01	7.79	7.79
	**L Shoulder**	13.03	14.59	14.59	14.59	14.59	14.69	18.79	13.03	13.03	13.25	15.52	10.46	10.46
	**L Elbow**	29.67	26.78	26.78	26.78	26.78	26.10	26.25	29.80	30.00	20.88	23.83	23.31	24.08
	**L Wrist**	47.36	48.28	48.28	48.28	48.28	47.89	39.96	36.96	39.46	45.51	36.07	35.11	34.08
	**R Shoulder**	12.17	14.46	14.46	14.46	14.46	13.88	15.34	12.19	12.17	13.10	12.33	9.51	9.50
	**R Elbow**	30.25	25.10	25.10	25.10	25.10	25.77	21.84	23.14	25.62	21.74	18.74	18.52	20.89
	**R Wrist**	50.67	48.14	48.14	48.14	48.14	44.44	35.46	34.90	36.83	42.59	32.80	32.04	32.69
	**L Hip**	10.27	12.70	12.70	12.70	12.70	12.86	10.26	10.27	10.27	10.24	8.52	8.52	8.52
	**L Knee**	22.62	20.78	20.78	20.78	20.78	21.22	19.87	19.83	20.43	16.85	16.28	16.19	16.45
	**L Ankle**	23.29	21.50	21.50	21.50	21.50	21.52	20.75	20.91	21.43	17.09	16.68	16.76	17.10
	**R Hip**	10.18	10.60	10.60	10.60	10.60	10.29	10.10	10.18	10.18	8.25	8.22	8.30	8.30
	**R Knee**	26.19	22.05	22.05	22.05	22.05	23.78	21.26	21.85	22.86	18.94	18.13	18.67	19.61
	**R Ankle**	22.73	20.47	20.47	20.47	20.47	20.54	19.06	19.55	21.02	15.97	15.37	15.68	17.02
	**Head**	10.88	13.17	13.17	13.17	13.17	16.50	11.87	10.88	10.88	17.79	10.27	9.51	9.51
**G6**	**Pelvis**	9.44	9.70	9.70	9.70	9.70	9.76	7.73	7.73	7.73	7.82	6.60	6.60	6.60
	**Spine Naval**	7.58	10.21	10.21	10.21	10.21	16.76	6.22	5.93	5.93	16.05	5.12	4.95	4.95
	**Neck**	9.94	11.63	11.63	11.63	11.63	15.41	8.53	8.46	8.46	14.67	7.28	7.32	7.32
	**L Shoulder**	15.42	15.74	15.74	15.74	15.74	15.76	19.43	14.07	14.07	13.71	15.65	10.87	10.87
	**L Elbow**	32.09	28.19	28.19	28.19	28.19	27.36	28.07	30.17	30.01	21.03	23.62	22.83	22.76
	**L Wrist**	52.02	47.37	47.37	47.37	47.37	47.48	40.25	38.60	40.94	43.70	35.32	34.64	33.91
	**R Shoulder**	14.67	15.11	15.11	15.11	15.11	14.73	15.81	13.14	13.14	13.81	12.04	9.82	9.82
	**R Elbow**	35.02	27.11	27.11	27.11	27.11	28.78	24.67	25.92	27.45	23.47	20.29	20.08	21.65
	**R Wrist**	57.06	49.82	49.82	49.82	49.82	46.99	38.69	37.46	38.60	41.80	34.96	33.83	33.92
	**L Hip**	11.09	11.87	11.87	11.87	11.87	12.39	9.44	9.44	9.44	9.59	7.51	7.51	7.51
	**L Knee**	23.70	19.43	19.43	19.43	19.43	19.76	18.66	18.68	18.92	15.40	14.75	14.76	14.92
	**L Ankle**	23.62	20.43	20.43	20.43	20.43	20.63	20.34	20.47	20.70	16.03	15.92	16.03	16.14
	**R Hip**	11.09	10.70	10.70	10.70	10.70	10.53	9.39	9.37	9.37	8.26	7.37	7.33	7.33
	**R Knee**	26.32	20.17	20.17	20.17	20.17	21.56	19.22	19.79	20.76	16.83	15.65	16.15	16.96
	**R Ankle**	22.28	19.23	19.23	19.23	19.23	19.22	18.00	18.57	19.77	15.03	14.37	14.64	15.82
	**Head**	11.64	12.77	12.77	12.77	12.77	15.84	10.46	10.27	10.27	16.32	8.95	8.91	8.91

## Appendix B

**Table A5:** RMSE result of the TNOS experiment.

	**Gesture 1**				**Gesture 2**				**Gesture 3**			
**TNOS**	**1**	**2**	**3**	**4**	**1**	**2**	**3**	**4**	**1**	**2**	**3**	**4**
**Pelvis**	43.80	34.68	31.57	**30.29**	52.05	39.36	35.07	32.71	53.12	45.37	42.58	**37.86**
**Spine Naval**	64.67	51.10	46.81	**45.15**	74.02	58.48	51.41	46.61	71.33	58.81	53.35	**48.22**
**Neck**	59.53	45.92	40.68	**38.69**	73.43	58.82	51.61	48.06	68.22	54.31	49.55	**44.71**
**L Shoulder**	64.22	48.42	38.55	**35.49**	78.30	61.09	46.22	40.78	72.95	55.90	44.72	**40.33**
**L Elbow**	72.43	56.64	40.52	**37.84**	98.83	75.85	51.71	45.59	112.68	85.58	56.37	**40.99**
**L Wrist**	106.31	93.77	63.82	**54.24**	147.66	125.14	83.78	69.29	188.05	166.06	113.39	**66.62**
**R Shoulder**	60.32	43.48	35.65	**31.80**	75.05	55.99	46.38	41.07	66.49	48.14	39.57	**33.42**
**R Elbow**	92.26	73.08	41.74	**33.28**	110.94	87.90	57.38	48.71	115.95	82.46	53.63	**36.15**
**R Wrist**	142.59	125.17	74.50	**54.34**	168.38	143.93	91.31	73.30	194.83	168.88	109.63	**56.75**
**L Hip**	51.22	39.51	35.02	**33.00**	56.22	43.30	37.82	34.92	56.68	48.15	44.55	**39.52**
**L Knee**	33.52	27.60	25.42	**24.34**	51.04	43.48	40.73	39.32	55.77	44.93	42.21	**37.96**
**L Ankle**	44.44	35.74	32.21	**30.37**	53.54	41.94	39.25	37.83	57.24	43.38	41.04	**38.22**
**R Hip**	47.06	35.12	31.11	**29.18**	54.97	41.30	35.54	32.02	54.99	46.00	42.47	**37.25**
**R Knee**	33.83	26.74	24.06	**22.50**	49.75	40.37	37.09	35.41	44.65	36.37	33.69	**29.14**
**R Ankle**	42.53	34.90	29.82	**28.17**	51.88	39.20	35.98	34.47	42.88	35.18	32.67	**30.36**
**Head**	60.00	46.02	39.84	**37.53**	74.96	60.28	53.17	49.43	69.08	54.47	49.78	**44.59**
	**Gesture 4**				**Gesture 5**				**Gesture 6**			
**TNOS**	**1**	**2**	**3**	**4**	**1**	**2**	**3**	**4**	**1**	**2**	**3**	**4**
**Pelvis**	53.74	44.74	38.61	**35.89**	50.12	38.76	34.66	32.88	51.00	39.88	36.24	**34.42**
**Spine Naval**	72.33	60.75	51.25	**48.76**	70.53	59.35	49.60	44.83	68.07	55.96	49.13	**46.03**
**Neck**	66.77	57.90	46.83	**43.37**	73.95	65.24	55.72	51.73	62.79	50.67	44.26	**42.05**
**L Shoulder**	72.27	60.80	46.95	**39.52**	76.95	69.59	51.91	42.51	70.41	55.48	43.27	**38.32**
**L Elbow**	94.83	73.07	49.00	**43.41**	109.30	102.77	66.25	53.35	84.44	63.12	42.88	**38.58**
**L Wrist**	139.50	108.09	71.74	**59.21**	165.96	142.33	87.54	73.26	121.91	99.03	62.03	**54.08**
**R Shoulder**	68.83	55.44	44.88	**38.37**	72.69	57.36	44.66	40.53	66.66	52.14	43.09	**38.70**
**R Elbow**	99.57	76.59	50.43	**42.35**	104.31	99.66	55.31	43.17	92.59	69.23	44.77	**39.57**
**R Wrist**	148.02	118.86	72.61	**56.74**	167.31	146.13	89.96	71.84	136.60	102.15	62.22	**50.91**
**L Hip**	59.64	50.55	41.92	**38.23**	54.16	42.47	37.38	35.30	58.08	45.33	39.10	**36.65**
**L Knee**	98.87	68.87	46.53	**43.14**	60.30	46.47	42.50	40.93	43.90	35.24	32.45	**31.26**
**L Ankle**	121.12	75.00	50.27	**47.06**	61.91	49.23	46.56	45.40	49.05	39.00	35.94	**34.50**
**R Hip**	56.38	46.67	39.53	**35.32**	53.04	40.10	34.54	32.02	54.49	43.01	37.46	**34.56**
**R Knee**	101.79	90.00	47.80	**40.77**	63.03	53.73	42.61	39.49	43.09	32.99	30.04	**28.45**
**R Ankle**	131.88	100.42	56.18	**48.80**	60.99	46.48	41.85	40.15	48.95	37.91	34.61	**33.13**
**Head**	67.08	58.79	47.15	**42.77**	76.87	67.56	58.87	55.18	63.26	50.79	44.32	**41.88**

**Table A6:** STD result of the TNOS experiment.

	**Gesture 1**				**Gesture 2**				**Gesture 3**			
**TNOS**	**1**	**2**	**3**	**4**	**1**	**2**	**3**	**4**	**1**	**2**	**3**	**4**
**Pelvis**	4.70	3.81	3.43	**3.17**	15.29	11.42	10.81	**9.94**	15.50	13.45	12.93	**9.76**
**Spine Naval**	8.84	6.80	6.55	**5.74**	14.08	12.82	12.61	**8.03**	15.41	13.74	12.96	**9.22**
**Neck**	13.17	9.70	9.08	**8.43**	18.46	14.51	13.13	**9.81**	20.01	15.84	14.72	**11.02**
**L Shoulder**	16.11	10.77	10.03	**9.09**	20.72	19.53	17.74	**14.14**	21.44	17.97	15.52	**12.62**
**L Elbow**	31.44	26.01	14.85	**12.96**	43.08	42.04	27.59	**22.49**	53.62	47.73	28.46	**17.56**
**L Wrist**	55.25	52.66	34.21	**26.18**	70.82	67.92	48.90	**37.40**	97.58	96.12	66.63	**31.50**
**R Shoulder**	16.32	11.46	9.63	**7.85**	21.13	18.36	16.95	**13.31**	19.68	16.12	13.73	**10.00**
**R Elbow**	42.65	38.43	19.27	**12.98**	50.99	51.77	33.90	**27.56**	53.15	46.10	28.77	**16.19**
**R Wrist**	73.10	71.78	45.43	**28.59**	80.44	77.89	58.60	**45.89**	95.39	96.91	69.11	**31.37**
**L Hip**	6.15	4.34	3.93	**3.40**	16.51	12.54	11.18	**9.78**	16.24	13.26	12.60	**9.45**
**L Knee**	6.55	5.34	4.77	**4.60**	20.31	17.13	16.15	**15.43**	24.39	15.97	14.57	**12.54**
**L Ankle**	8.70	6.77	6.02	**5.73**	22.90	16.42	15.57	**14.97**	24.66	14.54	13.65	**13.00**
**R Hip**	5.78	4.20	3.90	**3.59**	16.54	12.68	11.96	**10.93**	17.26	14.43	13.78	**10.73**
**R Knee**	5.89	4.79	4.45	**4.24**	20.72	17.72	16.39	**15.68**	17.49	14.73	13.93	**11.92**
**R Ankle**	9.88	7.85	7.06	**6.84**	24.65	17.25	15.81	**15.17**	17.63	13.77	13.06	**12.13**
**Head**	15.73	11.68	10.84	**10.10**	21.07	16.31	14.95	**11.57**	21.97	17.43	16.14	**12.29**
	**Gesture 4**				**Gesture 5**				**Gesture 6**			
**TNOS**	1	2	3	4	1	2	3	4	1	2	3	4
**Pelvis**	16.43	14.48	12.08	**10.21**	9.71	7.00	6.84	**6.60**	12.17	8.57	8.32	**7.59**
**Spine Naval**	17.87	19.26	16.67	**10.82**	10.74	7.99	8.70	**5.12**	11.29	9.20	9.98	**6.92**
**Neck**	18.83	20.09	15.84	**11.69**	12.89	12.13	11.02	**7.28**	14.79	12.61	11.36	**9.45**
**L Shoulder**	24.02	24.58	20.35	**15.03**	15.52	18.38	18.97	**15.65**	19.21	19.38	16.30	**13.70**
**L Elbow**	52.69	41.61	24.17	**19.71**	55.08	52.91	33.33	**23.62**	43.51	36.56	22.37	**19.31**
**L Wrist**	85.21	65.07	43.43	**33.24**	93.62	71.66	47.06	**35.32**	69.92	60.10	35.55	**28.42**
**R Shoulder**	22.14	20.98	17.54	**13.52**	16.60	16.43	13.93	**12.04**	20.17	16.30	15.35	**13.19**
**R Elbow**	52.03	44.36	26.50	**20.59**	50.31	53.38	29.81	**20.29**	46.98	38.48	22.63	**18.98**
**R Wrist**	83.31	75.96	45.99	**33.14**	86.59	67.04	48.64	**34.96**	74.64	59.49	36.43	**27.26**
**L Hip**	19.05	17.81	13.70	**11.36**	11.04	8.27	7.64	**7.51**	13.79	10.91	10.38	**9.22**
**L Knee**	64.29	39.28	20.62	**17.88**	27.80	17.14	15.41	**14.75**	15.75	11.12	10.30	**9.69**
**L Ankle**	80.83	40.33	22.53	**20.01**	27.02	17.54	16.45	**15.92**	19.08	14.40	13.15	**12.73**
**R Hip**	18.55	17.39	14.57	**11.83**	11.60	8.57	7.94	**7.37**	14.71	11.67	10.61	**9.71**
**R Knee**	65.09	60.97	23.33	**17.53**	31.09	25.05	17.91	**15.65**	16.16	11.49	10.56	**10.13**
**R Ankle**	87.84	64.80	28.85	**23.05**	28.56	18.02	15.36	**14.37**	21.07	15.32	13.67	**13.38**
**Head**	20.60	21.85	16.89	**12.70**	14.98	13.16	12.24	**8.95**	17.36	14.58	12.95	**10.88**

## References

[1] Ma M., Proffitt R., Skubic M. (2018). Validation of a Kinect V2 based rehabilitation game. PLoS ONE.

[2] Taha A., Zayed H.H., Khalifa M., El-Horbaty E.-S.M. (2015). Skeleton-based human activity recognition for video surveillance. Int. J. Sci. Eng. Res..

[3] Varshney N., Bakariya B., Kushwaha A.K.S., Khare M. (2021). Rule-based multi-view human activity recognition system in real time using skeleton data from RGB-D sensor. Soft Comput..

[4] Cippitelli E., Gasparrini S., Gambi E., Spinsante S. (2016). A human activity recognition system using skeleton data from RGBD sensors. Comput. Intell. Neurosci..

[5] Bari A.H., Gavrilova M.L. (2019). Multi-layer perceptron architecture for kinect-based gait recognition. Computer Graphics International Conference.

[6] Yao A., Gall J., Fanelli G., Van Gool L. (2011). Does human action recognition benefit from pose estimation?. Proceedings of the 22nd British Machine Vision Conference (BMVC 2011).

[7] Schlagenhauf F., Sreeram S., Singhose W. (2018). Comparison of kinect and vicon motion capture of upper-body joint angle tracking. Proceedings of the 2018 IEEE 14th International Conference on Control and Automation (ICCA).

[8] Shaikh M.B., Chai D. (2021). RGB-D Data-based Action Recognition: A Review. Sensors.

[9] Wang P., Li W., Ogunbona P., Wan J., Escalera S. (2018). RGB-D-based human motion recognition with deep learning: A survey. Comput. Vis. Image Underst..

[10] Liu B., Cai H., Ju Z., Liu H. (2019). RGB-D sensing based human action and interaction analysis: A survey. Pattern Recognit..

[11] Tölgyessy M., Dekan M., Chovanec Ľ., Hubinský P. (2021). Evaluation of the azure Kinect and its comparison to Kinect V1 and Kinect V2. Sensors.

[12] Romeo L., Marani R., Malosio M., Perri A.G., D’Orazio T. (2021). Performance analysis of body tracking with the microsoft azure Kinect. Proceedings of the 2021 29th Mediterranean Conference on Control and Automation (MED).

[13] Izadi S., Kim D., Hilliges O., Molyneaux D., Newcombe R., Kohli P., Shotton J., Hodges S., Freeman D., Davison A. (2011). KinectFusion: Real-time 3D reconstruction and interaction using a moving depth camera. Proceedings of the 24th Annual ACM Symposium on User Interface Software and Technology.

[14] Tölgyessy M., Dekan M., Chovanec Ľ. (2021). Skeleton Tracking Accuracy and Precision Evaluation of Kinect V1, Kinect V2, and the Azure Kinect. Appl. Sci..

[15] Aguileta A.A., Brena R.F., Mayora O., Molino-Minero-Re E., Trejo L.A. (2019). Multi-sensor fusion for activity recognition—A survey. Sensors.

[16] Gravina R., Alinia P., Ghasemzadeh H., Fortino G. (2017). Multi-sensor fusion in body sensor networks: State-of-the-art and research challenges. Inf. Fusion.

[17] Yeung L.-F., Yang Z., Cheng K.C.-C., Du D., Tong R.K.-Y. (2021). Effects of camera viewing angles on tracking kinematic gait patterns using Azure Kinect, Kinect v2 and Orbbec Astra Pro v2. Gait Posture.

[18] Kim Y., Baek S., Bae B.C. (2017). Motion capture of the human body using multiple depth sensors. Etri J..

[19] Colombel J., Daney D., Bonnet V., Charpillet F. (2021). Markerless 3D Human Pose Tracking in the Wild with fusion of Multiple Depth Cameras: Comparative Experimental Study with Kinect 2 and 3. Activity and Behavior Computing.

[20] Chen N., Chang Y., Liu H., Huang L., Zhang H. (2018). Human pose recognition based on skeleton fusion from multiple kinects. Proceedings of the 2018 37th Chinese Control Conference (CCC).

[21] Núñez J.C., Cabido R., Montemayor A.S., Pantrigo J.J. (2017). Real-time human body tracking based on data fusion from multiple RGB-D sensors. Multimed. Tools Appl..

[22] Wu Y., Gao L., Hoermann S., Lindeman R.W. (2018). Towards robust 3D skeleton tracking using data fusion from multiple depth sensors. Proceedings of the 2018 10th International Conference on Virtual Worlds and Games for Serious Applications (VS-Games).

[23] Desai K., Prabhakaran B., Raghuraman S. (2018). Combining skeletal poses for 3D human model generation using multiple Kinects. Proceedings of the 9th ACM Multimedia Systems Conference.

[24] Moon S., Park Y., Ko D.W., Suh I.H. (2016). Multiple kinect sensor fusion for human skeleton tracking using Kalman filtering. Int. J. Adv. Robot. Syst..

[25] Zhang H., He X., Liu Y. (2020). A Human Skeleton Data Optimization Algorithm for Multi-Kinect. Proceedings of the 2020 Asia-Pacific Conference on Image Processing, Electronics and Computers (IPEC).

[26] Ryselis K., Petkus T., Blažauskas T., Maskeliūnas R., Damaševičius R. (2020). Multiple Kinect based system to monitor and analyze key performance indicators of physical training. Hum. Cent. Comput. Inf. Sci..

[27] Swain M.J., Ballard D.H. (1992). Indexing via color histograms. Active Perception and Robot Vision.

[28] Fischler M.A., Bolles R.C. (1981). Random sample consensus: A paradigm for model fitting with applications to image analysis and automated cartography. Commun. ACM.

[29] Gower J.C., Dijksterhuis G.B. (2004). Procrustes Problems.

[30] Arun K.S., Huang T.S., Blostein S.D. (1987). Least-squares fitting of two 3-D point sets. IEEE Trans. Pattern Anal. Mach. Intell..

[31] Garrido-Jurado S., Munoz-Salinas R., Madrid-Cuevas F.J., Medina-Carnicer R. (2016). Generation of fiducial marker dictionaries using mixed integer linear programming. Pattern Recognit..

[32] Ester M., Kriegel H.-P., Sander J., Xu X. (1996). A density-based algorithm for discovering clusters in large spatial databases with noise. Kdd, 1996.

[33] Haller E., Scarlat G., Mocanu I., Trăscău M. (2013). Human activity recognition based on multiple Kinects. International Competition on Evaluating AAL Systems through Competitive Benchmarking.

[34] Kalman R.E. (1960). A new approach to linear filtering and prediction problems. J. Basic Eng..

[35] Naeemabadi M., Dinesen B., Andersen O.K., Hansen J. (2018). Influence of a marker-based motion capture system on the performance of Microsoft Kinect v2 skeleton algorithm. IEEE Sens. J..

[36] Naeemabadi M., Dinesen B., Andersen O.K., Hansen J. (2018). Investigating the impact of a motion capture system on Microsoft Kinect v2 recordings: A caution for using the technologies together. PLoS ONE.

